# Current *in vitro* Myocardial Infarction Co-Culture Models and their Verification for Clinical Relevance

**DOI:** 10.1017/erm.2026.10058

**Published:** 2026-06-15

**Authors:** Sara Lugmayr, Elisabeth Heuböck, David Bernhard, Markus Mandl, Verena Charwat

**Affiliations:** Department of Pathophysiology and Clinical Research Institute for Cardiovascular and Metabolic Disease, Faculty of Medicine, Johannes Kepler University, Linz, Austria

**Keywords:** cardiomyocytes, clinical relevance, co-culture, *in vitro* models, myocardial infarction models, translation

## Abstract

**Background:**

Myocardial infarction (MI) remains a critical focus in cardiovascular research, with *in vitro* models serving as essential tools for investigating pathophysiological mechanisms and evaluating therapeutic strategies. Despite their widespread use, the clinical relevance of these models is often assumed rather than rigorously validated.

**Methods:**

In this review, we systematically examined 28 studies from the last decade that employed *in vitro* co-culture models of MI, selected via a PubMed search according to pre-defined criteria. Each study was analysed with respect to ischaemia/reperfusion (I/R)-mimicking conditions applied, species and cell types used, and strategies for verifying clinical relevance.

**Results:**

Our analysis revealed considerable heterogeneity in experimental design. Protocols for inducing I/R stress and the duration of treatment varied widely between studies, underscoring a lack of methodological standardization. Cell sources included swine, rat, mouse and human-derived cells, with nearly half of the studies utilizing cross-species combinations – often without addressing the implications for translational validity. Verification strategies were inconsistently applied and frequently limited to basic endpoints such as cell viability and oxidative stress assays. Some studies also employed *in vivo* models to validate *in vitro* findings.

**Conclusions:**

To enhance translational potential, future studies should prioritize human-derived cells, standardized I/R protocols and verification strategies that better recapitulate MI pathology.

## Introduction

Myocardial infarction (MI) research holds significant socioeconomic and medical importance due to its high prevalence and substantial impact on global health. Cardiovascular diseases, including MI, remain the leading cause of mortality worldwide, accounting for an estimated 17.9 million deaths annually, with MI as a primary contributor (Ref. 1). Advancing MI research is essential to develop effective prevention, diagnosis and treatment strategies, reducing both mortality and associated socioeconomic strain (Ref. 2).

Ischaemia–reperfusion (I/R) models are crucial for understanding the pathophysiological mechanisms underlying MI. These models simulate the transient loss of blood flow (ischaemia) followed by restoration (reperfusion), mimicking the clinical events of MI. I/R models provide valuable insights into oxidative stress, inflammation and cell death pathways, facilitating the development of cardioprotective therapies. For instance, they have been instrumental in identifying key therapeutic targets, such as reactive oxygen species (ROS) scavengers and anti-inflammatory agents. Moreover, these models help evaluate novel interventions like ischaemic preconditioning and post-conditioning. Their translational relevance is underscored by their role in bridging experimental findings with clinical outcomes in MI management (Refs 3, 4).

This review focuses on *in vitro* co-culture models, which are particularly relevant in I/R research as they allow the controlled study of cell–cell interactions under pathological conditions. Depending on their exact configuration, these models can replicate the cellular microenvironment of the heart, including the interplay between cardiomyocytes (CMs), endothelial cells (ECs), fibroblasts (FBs) and immune cells, which are all critically involved in I/R injury. Co-culture systems provide insights into how paracrine signalling, inflammatory mediators and oxidative stress contribute to tissue damage and repair processes. They also facilitate high-throughput screening of therapeutic interventions, thereby speeding up research, reducing reliance on animal models and improving translational accuracy (Refs 5, 6). While 2D cultures are cost-effective, they lack the complexity of cardiac tissue (Ref. 7). Advanced 3D models, like cardiac spheroids, engineered tissues and organoids (Ref. 8), better replicate the structure and function of the heart (Ref. 9).

Modelling I/R *in vitro* is a complex endeavour due to the multifaceted and dynamic nature of the underlying pathophysiology. Ischaemia refers to the restriction of blood supply and, consequently, oxygen and nutrient deprivation, while reperfusion represents the restoration of circulation. These phenomena trigger a cascade of cellular and molecular events, including oxidative stress, inflammation and metabolic dysregulation, which are difficult to replicate accurately in a controlled laboratory environment (Refs 5, 10). Additionally, the intricate cellular interactions involved in I/R injury *in vivo* present significant challenges for extrapolating findings from reductionist *in vitro* models (Ref. 11).

Investigating the validation status and relevance of current *in vitro* I/R co-culture models is therefore crucial, as this field is still in its infancy. Despite their promise in replicating cellular interactions and microenvironments, most co-culture systems lack comprehensive validation against *in vivo* and clinical outcomes, raising concerns about their translational applicability. Verification of these models is necessary to ensure that they accurately mimic key aspects of I/R pathology, such as oxidative stress, inflammatory signalling and tissue remodelling. Without standardized validation criteria, there is a risk of overestimating their predictive value, potentially leading to unreliable preclinical findings (Refs 6, 12). Addressing these gaps will enhance their utility in developing effective therapeutic strategies.

In this review, we set out to assess the current status of *in vitro* I/R co-culture models and specifically look at reported strategies and results of model verification and validation.

## Methods

In an effort to increase reproducibility and reduce author bias, we leaned on systematic review strategies in finding and screening literature for this review. We addressed the questions: *What current co-culture in vitro models of MI are available and how are they verified regarding clinical relevance?* Our literature pool is based on a defined PubMed search query (see next) including only original research articles involving co-culture models. We specifically excluded purely animal-based studies such as *in vivo*, *ex vivo*, Langendorff and Hetcam models as well as other non-cell culture models.

The search query was phrased as follows and submitted to PubMed on October 23^rd^, 2024:

((((‘cardiomyocytes’[All Fields] OR (‘cardiac’[All Fields] AND ‘myocytes’[All Fields]) OR ‘cardiocytes’[All Fields]) AND (‘vitro’[All Fields] OR (‘model’[All Fields] AND ‘system’[All Fields]) OR ‘model’[All Fields])) NOT ‘Review’[Publication Type]) NOT (‘ex vivo’[Title] OR ‘Langendorff heart’[Title] OR ‘isolated perfused heart’[Title] OR ‘het-cam’[Title])) AND (‘co-culture’[All Fields] OR ‘coculture’[All Fields] OR ‘co-culture’[All Fields])

The publication time range was restricted to the last decade: 2014 to 23^rd^ of October 2024.

Overall, these criteria yielded 270 publications, which were manually pre-screened based on their abstracts and methods to determine their primary focus. The resulting list of 62 articles focussing on MI were further assessed. Articles clearly out of scope for this review were eliminated (e.g., other focus than MI, no co-culture model, I/R modelled *in vivo* only): 27 articles were omitted because I/R was not modelled *in vitro* and four publications were excluded since the methods did not incorporate co-culture. Additionally, one study was excluded due to insufficient description of applied methods, leading to 30 publications passing our inclusion and exclusion criteria. Notably, two studies were additionally excluded in this review, namely, Siegel et al. and Maeda et al. Siegel et al. focussed on microvesicle-mediated effects of MI on liver cells and used co-culture of THP-1 and HepG2 cells (Ref. 13). Since this study primarily focussed on the liver, it was excluded. Maeda et al. focussed on rescue mechanisms for cardiomyocytes, using endometrial mesenchymal cells to show mitochondrial transfer in co-culture (Ref. 14). Due to the spatial separation between these two cell types *in vivo*, this publication was omitted. Thus, a final set of 28 publications was analysed and evaluated in this review. The selection process is illustrated in Figure 1.

**Figure 1.**
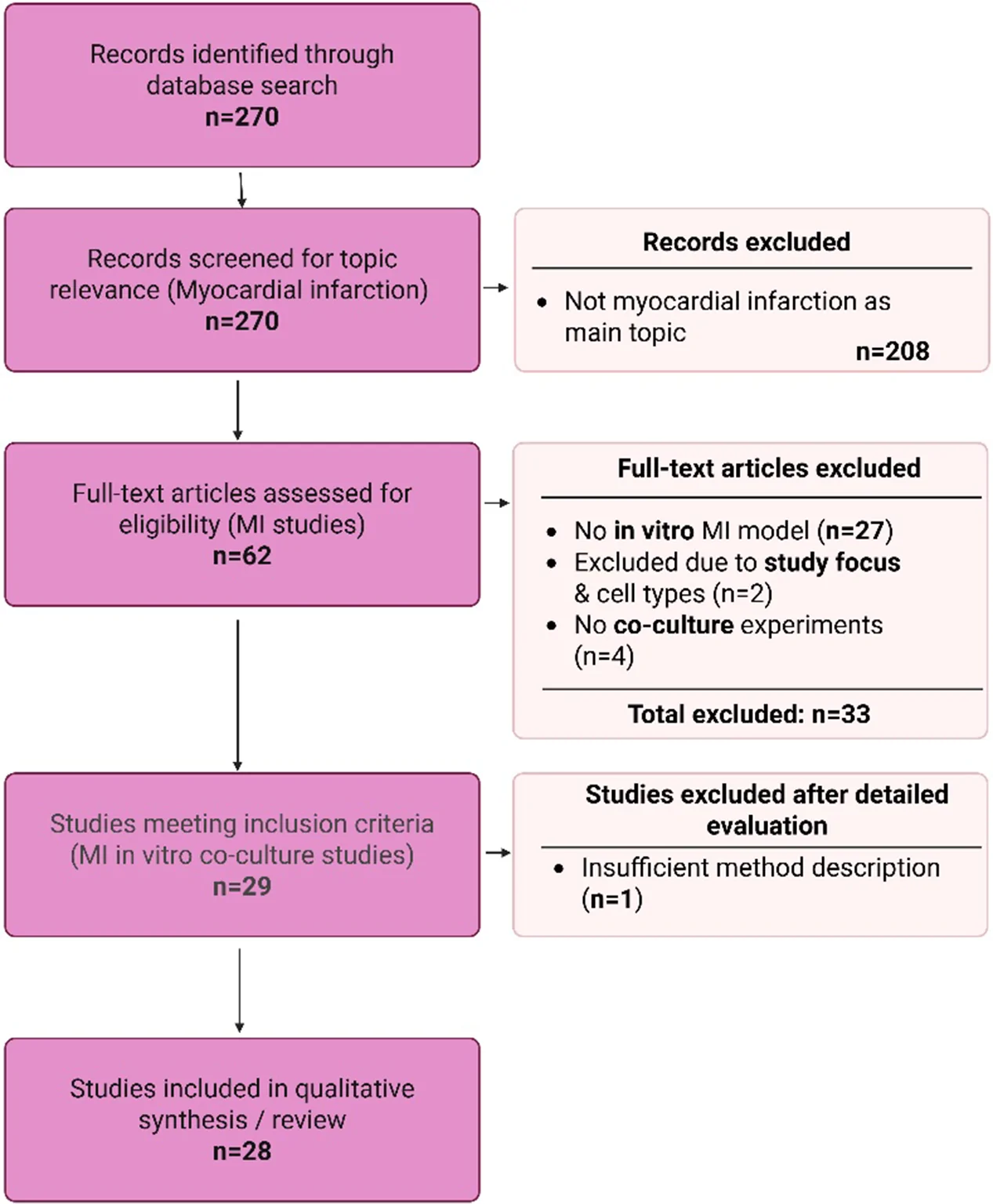
PRISMA style flow chart of the literature screening process. Figure 1. long description.

For addressing our research question ‘*What current co-culture* in vitro *models of MI are available and how are they verified regarding clinical relevance?*’ we assessed each publication regarding the following key aspects:

- Conditions applied to mimic ischaemia and/or reperfusion injury
- Cell species and types used to generate the *in vitro* model
- Strategies and biomarkers used to evaluate and verify the experimental data

## Results

### Approaches to create I/R conditions *in vitro*

We were interested to see whether a common set of parameters to model I/R can be identified from the reviewed publications. Establishing a standardized protocol would facilitate assessment of clinical relevance and help future inter-study comparability. We compared the I/R approaches and parameters between the analysed papers to determine whether a specific procedure is generally recognized as the most efficient and reproducible model. A concise overview of the I/R conditions used in each study is provided in the Supplementary Materials (Table S1).

The majority of authors included hypoxia as stressor of mimicking ischaemia, with only three papers solely utilizing nutrient deprivation (Refs 15, 16, 17). Most commonly, a mixture of serum-deprivation and hypoxic conditions were applied. Intriguingly, both duration of hypoxia treatment and oxygen concentrations varied greatly. Figures 2 and 3A depict applied oxygen levels and hypoxia durations. Two studies (Refs 18, 19) used multiple hypoxia levels, therefore, 27 data points (from 24 publications) are shown in in Figure 2. Three studies also evaluated multiple treatment durations under hypoxic conditions (Refs 16, 19, 20), and therefore, 30 data points are presented in Figure 3A.

**Figure 2.**
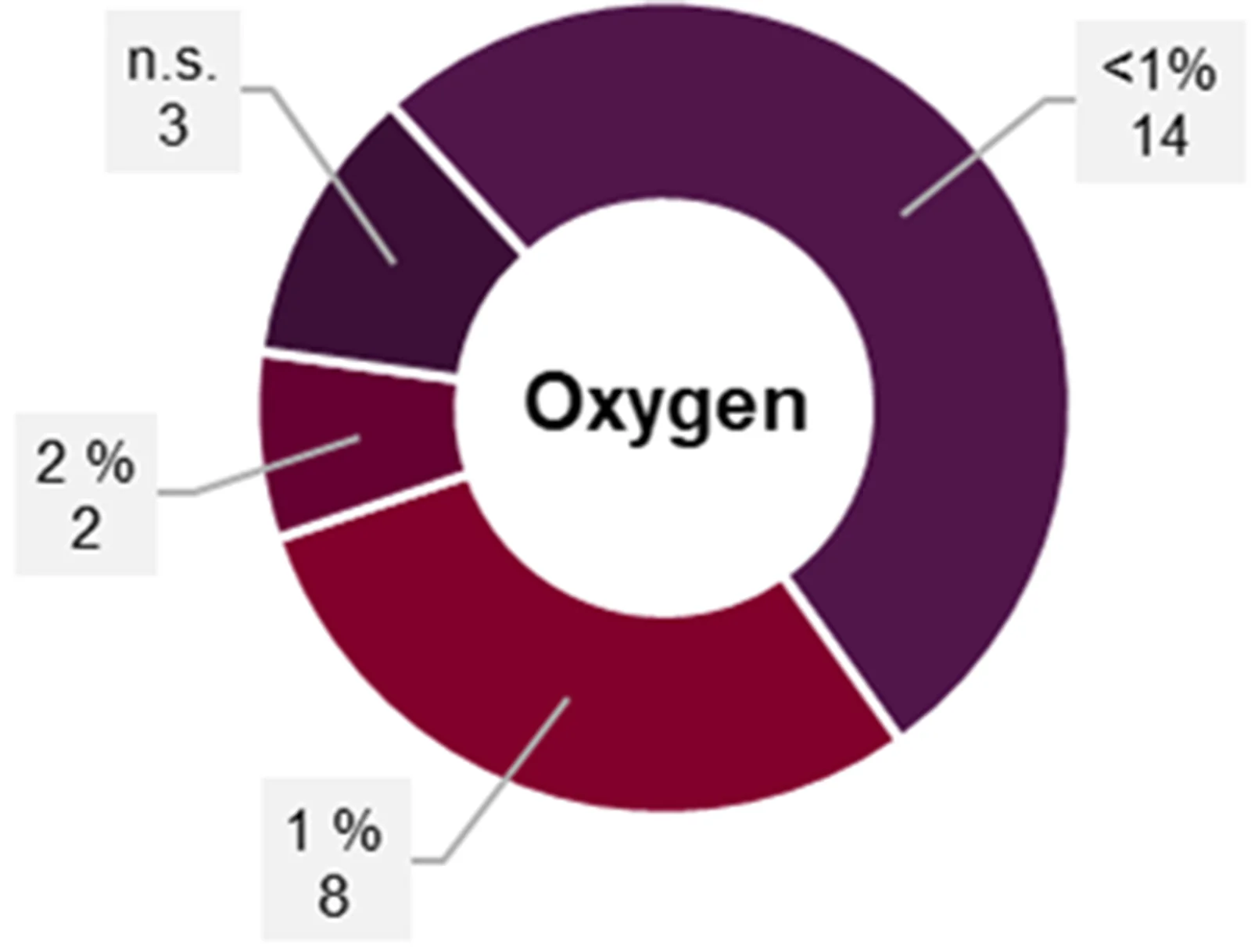
Oxygen concentrations applied during ischaemia simulation. Number of studies using the respective concentration range are stated below the oxygen percentage. n.s.: not specified.

**Figure 3.**
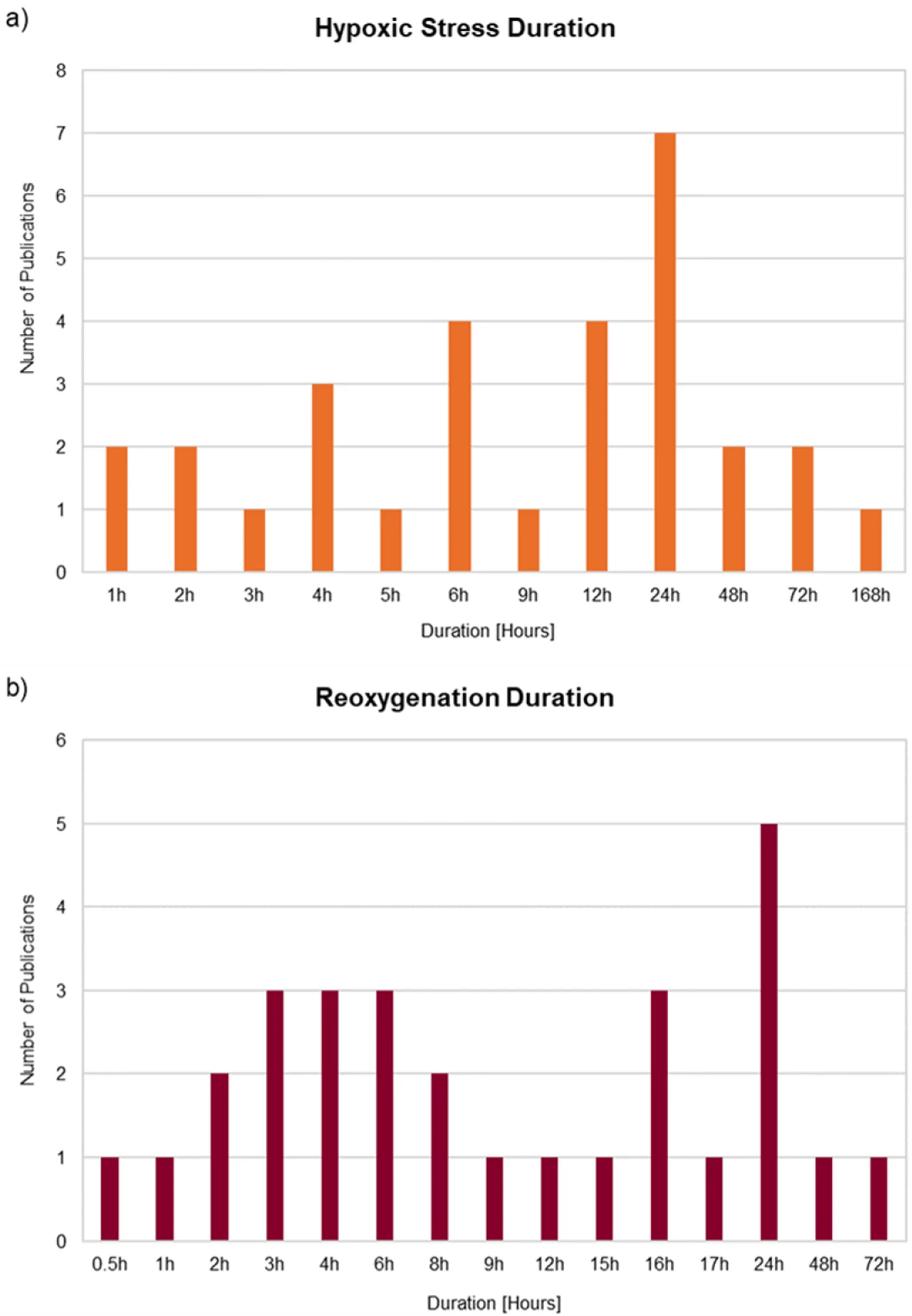
Applied time intervals for mimicking ischaemia and reperfusion stress. **A.** Treatment duration under hypoxic conditions. If multiple durations were used in the same publication, all timeframes were included in this graph. **B.** Duration of Reoxygenation Stress Treatments. If multiple durations were used in the same publication, all timeframes were included in the graph. Figure 3. long description.

Strikingly, only four publications provided a rationale for their respective choice of hypoxia conditions: Zhang et al. determined the optimal hypoxia conditions based on the analysis of survival, early apoptosis and late apoptosis ensuring an appropriate level of damage that is sufficient but not excessive, in line with the characteristics of I/R injury at 0.1% O_2_ (Ref. 21). Hafez et al. defined the ideal hypoxic duration as the time point, where 100% of the cells expressed HIF-1α (Ref. 20). Aonuma et al. selected 0% O_2_ as the best condition by visualizing neonatal rat ventricular myocyte (NRVM) apoptosis (Ref. 19). Yang et al. selected 12 h of 0.5% O_2_ as their hypoxia conditions based on literature data and comparison of six models (Ref. 22).

In addition to reduced oxygen supply, nutrient deprivation was a common approach to model the ischaemia phase. Typically, this was realized by removal of glucose or serum from the regular culture media or by applying a special ischaemia media formulation. Additionally, several authors mimicked I/R stress through treatment with special agents, such as H_2_O_2_ or CoCl_2_. Again, the exact conditions varied greatly between studies and are listed in Table 1.

**Table 1.** Media compositions and treatments to simulate I/R stress *in vitro* Table 1. long description.

Condition	Media composition
Glucose deprivation	0% glucose DMEM (Refs 22, 23) Glucose deprivation before hypoxia (Ref. 24)
Serum deprivation	Serum-free medium before co-culturing with stem cells (Ref. 15) Overnight serum-starvation (Ref. 22) Serum-free EGM-2 medium (Ref. 16)
Combined approaches (glucose and serum deprivation)	Glucose- and serum-free DMEM-F12 (Refs 21, 25) Serum-free, glucose-free, DMEM (Refs 20, 22, 26) Serum-free DMEM/low (1000 mg/L) glucose (Refs 22, 26) Glucose- and serum-free culture medium not specified (Ref. 27)
Specialized buffers/media	Ischaemic mimetic solution with precise ionic concentrations (Ref. 28) 2-deoxyglucose and sodium dithionite solution (Ref. 17) IMDM with β-mercaptoethanol and interferon-γ (Ref. 29) M199 with penicillin–streptomycin (Ref. 30) DMEM with 10% serum, maintaining partial nutrients (Ref. 31)
Other I/R inducing agents and treatments	CoCl_2_-containing medium (Ref. 32) 600 μM H_2_O_2_ (Ref. 33) 1 μM Doxorubicin (Ref. 33) Paraffin overlay (Ref. 17) Overlay with fibroblast layers (Ref. 34)

*Abbreviations:* DMEM: Dulbecco’s Modified Eagle’s Medium; EGM-2: endothelial growth medium-2; IMDM: Iscove’s Modified Dulbecco’s Medium.

A notable proportion of the analysed publications (18 of 24) did not solely focus on mimicking ischaemia but also examined the effects of reperfusion *in vitro* (Refs 17, 20–23, 25–28, 30, 31, 32, 35–40). Generally, reoxygenation during the reperfusion phase was modelled simply by setting the oxygen concentration back to ambient values. To simulate the end of nutrient deprivation, most authors replaced nutrient-deprived buffers with standard cell culture media supplemented with serum, glucose and other essential nutrients. Additionally, to further mimic the oxidative stress present during the reperfusion phase *in vivo*, oxidizing agents such as hydrogen peroxide (H_2_O_2_) were applied during the reperfusion phase (Ref. 35). Major variations were observed in the timeframes used to simulate reperfusion stress, as displayed in Figure 3B.

Another essential parameter for *in vitro* I/R models is the timing ischaemia and reperfusion phases. Clinically, a shorter time to treatment upon an ischaemic event reduces cardiac damage and improves patient outcome with recommended time-to-treatment of 2 h post-ECG diagnosis (Ref. 41). However, time between MI and treatment varies greatly, with a study reporting median times ranging from 1 to 24 h (Ref. 42). The reviewed *in vitro* hypoxia models, ranging from 1 to 168 h (commonly 24 h), reflect this clinical variability, which additionally complicates direct comparisons across studies.

Most authors conducted analysis directly after finishing the ‘reperfusion’ step. Yue et al. included 2 h of normoxic standard culture conditions after applying the 16-hour reperfusion stress treatment using H_2_O_2_ (Ref. 35). No rationale for this experimental approach was provided. Sebastião et al. aimed to obtain a regenerative curve by analysing their model before injury, directly after ischaemia and 1, 3, 8 and 16 h after onset of reperfusion conditions (Ref. 28).

We found that the general approaches to mimic I/R were similar between the publications: ischaemia was generally modelled via low oxygen and nutrient restriction, while reperfusion was mostly characterized by restoration of standard culture conditions. However, the protocol details including oxygen concentration, duration of ischaemia and reperfusion phase, and media composition varied greatly between models. Overall, we could not identify a common or optimized set of I/R model parameters.

### Cells and co-culture used in I/R models

#### Cell species

Interspecies differences are a major barrier to translating findings from animal models to human clinical applications (Ref. 43). Therefore, species origin should be carefully considered in *in vitro* studies as well. However, this aspect is often overlooked, and translatability across species is rarely addressed. To evaluate this in the context of our review, we analysed the species of origin used in the I/R *in vitro* models (see also Supplementary Table S2). Overall, cells from four species were utilized, with rodent cells being the most common. Among these, rat cells (18 out of 24 studies) (Refs 15, 17, 18, 19, 22, 25, 26, 27, 31, 32, 34–40, 44, 45) were used thrice as often as murine cells (6 out of 24) (Refs 19, 23, 29, 31, 34, 44). Cells of human origin were used in 13 studies (Refs 15–18, 20, 21, 22, 24, 28, 30, 33, 35, 45), while only one publication included swine cells in their experiments (Ref. 38). Of the 28 publications, 16 exclusively used cells from a single species (Refs 16, 20, 21, 23–30, 32, 33, 36, 37, 39), whereby human (Refs 16, 20, 21, 24, 28, 30, 33) and rat cells (Refs 25, 26, 27, 32, 36, 37, 39) were the most frequently used, followed by murine cells (Refs 23, 29) and swine cells (Ref 38) as depicted in Figure 4A. One publication was not included in Figure 4, as the authors did not state the species of all cell types used (Ref. 40). The remaining 11 publications combined cells from different species. The most common combination was human and rat cells (6 out of 24 studies) (Refs 15, 17, 18, 22, 35, 45), while murine and rat cells were combined in four studies (Refs 19, 31, 34, 44) and one study involved swine and rat cells (Ref. 38). Notably, human and murine cells were not combined in the evaluated papers.

**Figure 4.**
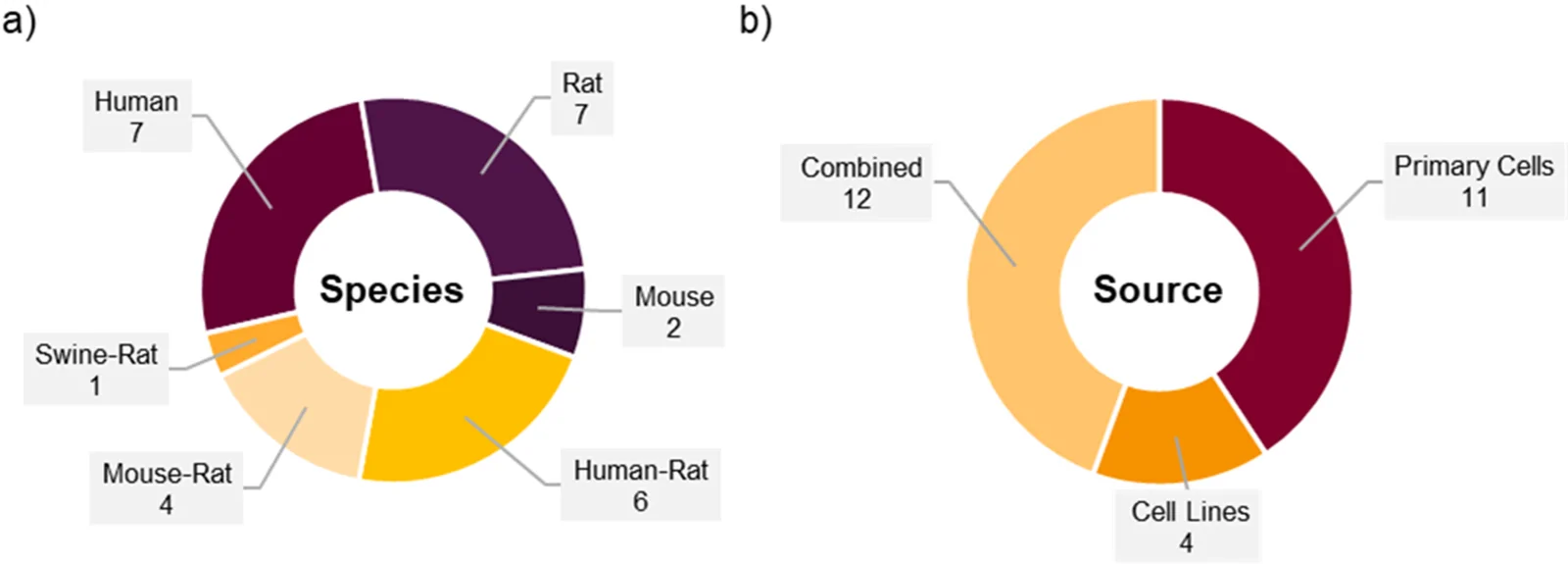
Species and cell sources for generation of *in vitro* models. **A.** Graph depicts cell species and their combinations used, with the number of publications incorporating the respective species or combination stated below. **B.** Graph shows the distribution of primary cells and cell lines used for *in vitro* model generation and the number of publications implementing them stated below. Figure 4. long description.

Although 11 studies combined cells from different species, only three of them addressed this issue directly (see also Supplementary Table S2). Yang et al. evaluated existing I/R models and combined species to reproduce the protocols (Ref. 22). Two groups stated difficulties with primary cells as the reason for species combination. Mosadegh et al. incorporated 3T3 cells (a murine fibroblast cell line) in their cardiomyocyte-fibroblast co-culture system due to low availability of primary cardiac fibroblasts and combined them with primary neonatal rat heart cells (Ref. 34). Similarly, the group of Kongpol et al. exclusively relied on cell lines, namely, EA.hy926 (human umbilical cord cell line) and H9C2 (rat ventricular cell line), stating non-specified ‘problems with primary cells’ as their rationale (Ref. 17). Yue et al. justified the combination of cellular species with the questionable maturity and functionality of human-induced pluripotent stem cell (iPSC)-derived CMs (iCMs) and the better characterization of neonatal rat CMs (Ref. 35). The combination of human iPSC-derived EC (iECs) and rat CMs was also beneficial for the subsequent analysis, since mRNAs could be easily assigned to the respective cell type, resulting in a more detailed transcriptome analysis.

#### Cell sources

Among the analysed papers, almost all used primary cells (23 out of 28 studies) (Refs 15, 16, 18, 19, 20, 22, 23, 26–39, 44, 45), although only 11 exclusively relied on primary cells (Figure 4B). In 12 publications, primary cells and cell lines, including iPSCs, were combined (Refs 18, 22, 25, 26, 28, 32–36, 38, 45) and four studies exclusively used cell lines and/or iPSC-derived cells (Refs 17, 21, 24, 25). Out of the four publications including the use of human iPSCs (hiPSCs) (Refs 21, 24, 28, 35), the group of Peters et al. solely relied on the usage of hiPSC-derived cells (Ref. 24).

#### Cell types

While all papers modelled I/R, the specific research focus varied. Hence, different cell types were selected to generate the models. Figure 5 illustrates the distribution of cell types for three of the four species used, whereby the cell sources are indicated by colours (primary cells = purple shades, cell lines = orange shades, human iPSC-derived cells = blue shades). Since only one publication used primary swine mesenchymal stem cells (MSCs), this species is not represented in the graph.

**Figure 5.**
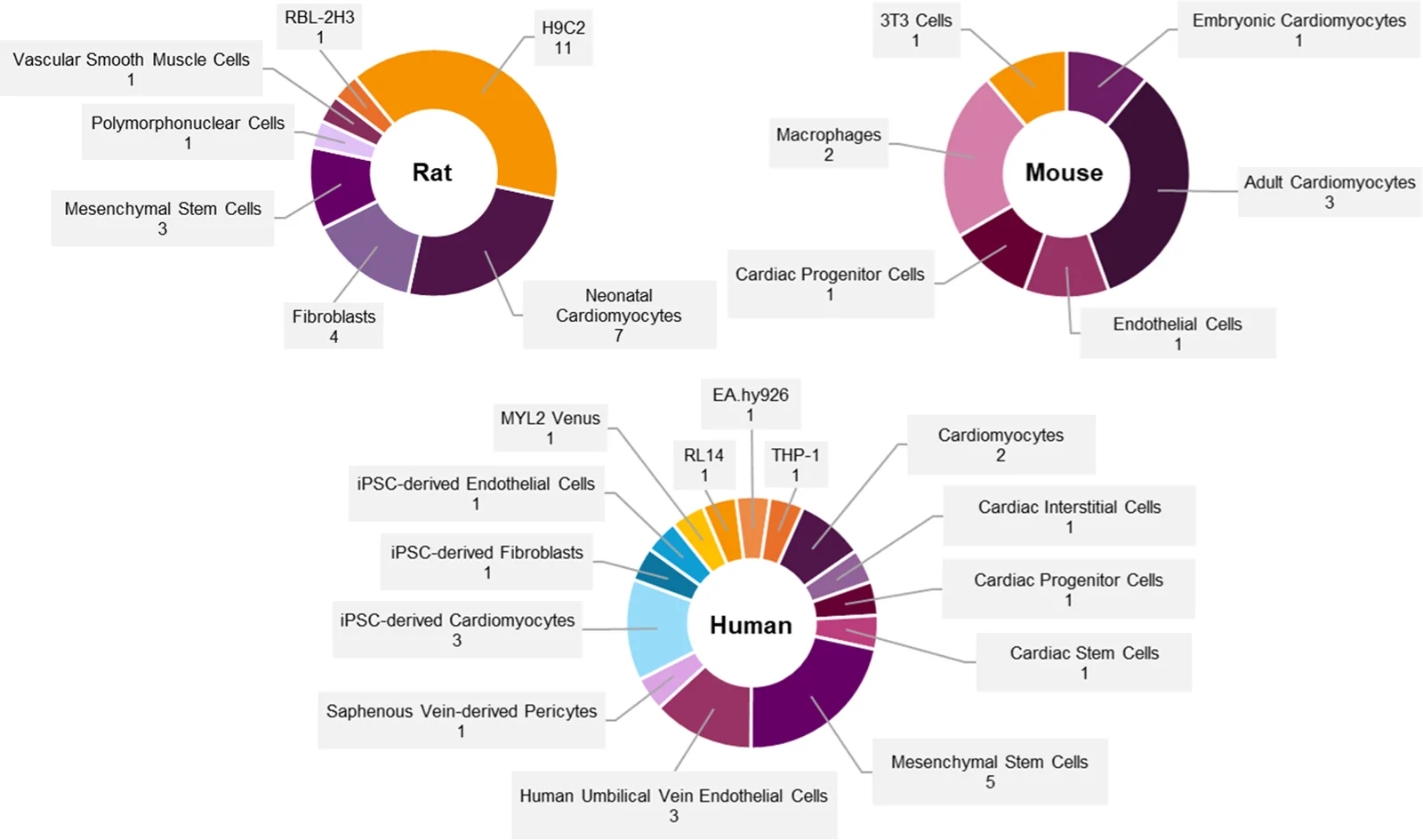
Cell types from rat, mouse and human used for *in vitro* model generation. Top left: rat cells; top right: murine cells, bottom: human cells; purple shades: primary cells; orange shades: cell lines; blue shades: induced pluripotent stem cell-derived (iPSC) cells. Numbers of publications including the respective cell types are included in the labels below the cell type name. EA.hy926: human umbilical vein cell line; H9C2: subclone of BD1X rat heart derived cell line; MYL2 Venus: embryonal stem cells myeloid line 2; RBL-2H3: rat basophilic leukaemia mast cells; RL14: human foetal ventricular cardiomyocyte cell line; THP-1: human leukaemia monocytic cell line; 3T3 cells: murine fibroblast cell line. Figure 5. long description.

Incorporated cell types fall into four main groups: cardiac cells, vascular cells, immune cells and mesenchymal cells. As expected, based on our literature search query, most models included cardiac cells. Cardiac cells included CMs/iCMs, cardiac interstitial, progenitor and stem cells, as well as cardiac cell lines, such as RL14. Overall, cardiac cells were mostly derived from primary tissues, except for cardiac rat cells (Figure 5, top left), where the cell line H9C2 (11 publications) (Refs 17, 18, 22, 25, 26, 27, 32, 36, 38, 40, 45), an embryonic ventricular myoblast cell line, was used most frequently followed by neonatal cardiomyocytes (7 studies) (Ref 15, 19, 31, 34, 35, 37, 44). Notably, all studies involving rat cell lines used the H9C2 line.

Vascular cells represent the second largest group and include ECs/iECs, human umbilical vein cells (HUVECs) and human saphenous vein-derived pericytes (SVPs). In the group of mesenchymal cells, FBs from all three species and from all cell sources (primary cells, cell lines and iPSC-derived FBs) were used. Additionally, primary swine, rat and human MSCs, mostly derived from bone marrow, were also incorporated in multiple experiments. The smallest portion was represented by immune cells, including murine macrophages (Refs 29, 31), polymorphonuclear cells (Ref. 39), RBL-2H3 cells (Ref. 37), a basophilic rat leukaemia cell line, and THP-1 cells (Ref. 21), a human monocyte cell line.

Overall, the greatest variety of cell types was observed for human cells (14 cell types), while seven rat and six murine cell types were used.

#### Co-culture methods

Out of the 28 analysed papers, only three publications incorporated three-dimensional approaches for the *in vitro* cell culture systems (Refs 21, 34, 35). The other publications relied on 2D culture, whereby transwell assays (Refs 17, 22, 23, 25, 27, 28, 31, 36, 38, 39, 40, 45) and 2D culture with ratio-based combination of cell types (Refs 15, 18, 19, 20, 24, 26, 29, 37) were used nearly equally by 12 and 9 studies, respectively. In four publications, both co-culture methods (transwell and ratio-based) were applied (Refs 16, 30, 33, 44). The high number of transwell assays can partly be explained by publications specifically addressing paracrine signalling as their research focus (Refs 17, 23, 28, 35, 39, 45). Supplementary Table S2 provides information on the culture system used in each study.

### Methods and biomarkers to verify clinical relevance

The translational applicability of an *in vitro* model to the *in vivo* physiology is a critical determinant of its relevance and scientific utility. To determine how translational aspects were addressed across the reviewed studies, we systematically evaluated the methodologies and biomarkers employed to relate I/R injury and treatment effects to clinically established endpoints. We grouped the biomarkers into three categories: (1) *stress response analyses*, encompassing assessments of cell viability, apoptosis and oxidative stress parameters; (2) *comparative evaluation with* in vivo *models*, serving to validate *in vitro* findings within a physiological context; and (3) *references to clinical and preclinical literature*, wherein authors aligned their experimental results with known clinical outcomes or therapeutic paradigms, thereby providing an interpretative framework for translational relevance.

#### Viability/apoptosis assays and oxidative stress assessment

The most frequently utilized parameters for validating *in vitro* MI models were cell viability and oxidative stress, which are often closely interconnected (Ref. 46). However, while these markers are necessary, they are not sufficient for accurately characterizing an I/R injury model. An I/R model that does not exhibit oxidative stress and loss of viability fails to adequately replicate the clinically observed myocardial damage. Conversely, viability and oxidative stress alone are highly nonspecific and provide limited biological significance, particularly if the extent of cell death or oxidative stress is not quantitatively correlated with the *in vivo* pathophysiology (Ref. 11).

As shown in Figure 6A, viability staining was a widely used method to assess effectiveness of I/R injury treatments. Viability was either assessed directly by labelling of dead or living cells (e.g., Annexin V/PI staining, fluorescent live/dead assay kits, Calcein AM, Trypan Blue) or indirectly by assessing caspase activity as a marker for apoptosis. Oxidative stress (Figure 6B) was typically detected via ROS indicator dyes or, indirectly, via the hypoxia-inducible factor HIF1-α. Some studies used unspecific tests such as LDH and MTT assays to evaluate oxidative stress responses. These assays provide a measure of metabolic activity and viability and are influenced by numerous factors including but not limited to oxidative stress. Despite this limitation, these assays were categorized under ‘oxidative stress assessment’ in our analysis (Figure 6B), since this is the context stated in the respective studies.

**Figure 6.**
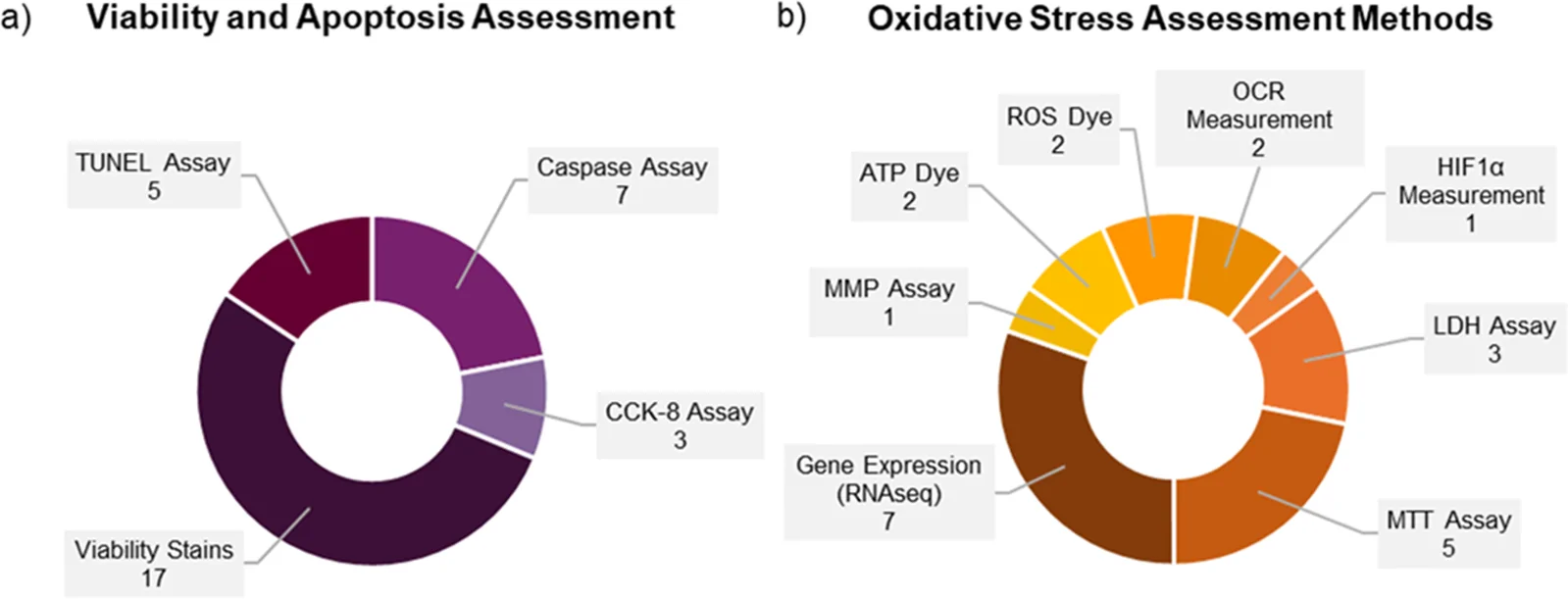
Biomarkers to verify clinical relevance *in vitro*. **A.** Viability and apoptosis assays: Number of studies using different classes of apoptosis and viability assays. Viability stains: for example, Calcein AM, Annexin V/PI, Trypan Blue. TUNEL assay: TdT-mediated dUTP nick end labelling assays. Caspase assay: cleaved caspase analysis of apoptosis. CCK-8 assay: Cell-Counting-Kit-8 for live cell quantification. **B.** Oxidative stress assessment: ATP: adenosine-triphosphate; HIF-1α: peroxisome-proliferator-activated-receptor (PPAR)-γ–hypoxia-inducible-factor-(HIF)-1 α; LDH: lactate dehydrogenase; MMP: mitochondrial membrane potential; MTT: 3-(4,5-dimethylthiazol-2-yl)-2,5-diphenyl-2H-tetrazolium bromide; ROS: reactive oxygen species; OCR: oxygen consumption rate. Figure 6. long description.

#### Direct comparison of *in vitro* findings with *in vivo* data

Despite ongoing efforts to reduce animal models, they are still a central and vital tool for preclinical testing. A relevant proportion of the *in vitro* studies assessed in this review were validated through direct comparison with *in vivo* experiments.

*In vivo*, the gold-standard model for MI involves surgical ligation of the left anterior descending (LAD) coronary artery in rats or mice, followed by removal of the ligation after a defined time period to induce I/R injury (Ref. 47). This procedure was employed in several of the studies included in this review (Refs 16, 18, 19, 33, 44, 45). In these studies, *in vivo* models were primarily used to validate findings obtained from *in vitro* experiments, particularly in the context of testing cell-based therapies or nanoparticle interventions. Specific examples include the intramyocardial administration of silica nanoparticles (Ref. 18), genetically modified cardiac progenitor cells (Ref. 19), mesenchymal stromal cells (MSCs) preconditioned with chloroquine (Ref. 16), or combinatorial injections of pericytes and cardiac stem cells (Ref. 33). Wang et al. investigated the regenerative potential of cardiomyocytes following MI through adenoviral-mediated overexpression of Connexin-43 (Ref. 44), while Huang et al. evaluated the therapeutic effects of exosomes derived from Atorvastatin-pretreated rat MSCs in infarcted murine hearts (Ref. 45). To assess therapeutic efficacy, the included studies employed a range of analytical markers, which are compared in Table 2. Notably, there appeared to be a general tendency to first demonstrate the beneficial effects of a given therapeutic approach *in vitro*, thereby establishing a mechanistic basis for subsequent *in vivo* studies.

**Table 2.** Applied *in vitro* and *in vivo* markers, in comparison Table 2. long description.

*In vitro* markers	*In vivo* markers	Authors
RT-qPCR for cardiac markers H_2_O_2_-induced ROS and apoptosis assay Western blot for pro-inflammatory cytokines TUNEL assay	Histology (Masson’s trichrome staining, α-SA-stain, CD-31 stain) Immunofluorescent labelling of infiltrating macrophages	Aonuma et al. (Ref. 19)
Immunofluorescence microscopy (Connexin-43, LC3, endosome, lysosome labelling) FACS (Connexin-43 surface expression, CD90) Cell adhesion and detachment assays Dye transfer assays Western blot (LC3, p62/SQSTM1)	Histology combined with immunofluorescence microscopy (labelled silica-nanoparticles in heart – engraftment assessment of human MSCs into infarcted mouse hearts; α-actinin staining)	Popara et al. (Ref. 18)
FACS (Mitotracker for MSC exchange, apoptosis dye, protein expression levels) Oxygen consumption rate measurement Transmission electron microscopy Measurement of heme oxygenase activity Immunocytochemistry (HO-1, mTFA, human mitochondria) RT-qPCR (PGC1a, mTFA)	Troponin T in mouse serum Western blot (Caspase-3) RT-qPCR (PGC1a, mTFA)	Mahrouf-Yorgov et al. (Ref. 33)
FACS (CD34, CD31, CD45, CD105, CD44 surface expression) Immunofluorescence (Oct-4, Sox-2, Nanog, c-Kit, PDGFRb, NG2, CD31, a-SMA, a-SA, Connexin-43, MHC) RT-qPCR (Tbx5, Connexin43, Cardiac Troponin T) EdU proliferation assay TUNEL assay Caspase 3/7 assay BrdU assay Migration assay *In vitro* Matrigel assay	ECG and hemodynamic measurements (left ventricular thickness, left ventricular ejection fraction, fractional shortening, ventricular pressure) Immunohistochemistry (a-Actin, CD34, DAPI) TUNEL assay Infarct size and myocardial fibrosis assessment Cardiomyocyte hypertrophy and nuclear density measurement	Avolio et al. (Ref. 16)
Time-lapse imaging with fluorescent nucleus labelling Calcium transients of CMs	Immunostaining ECG Infarct size analysis	Wang et al. (Ref. 44)
FACS (Annexin V/PI) Matrigel-tube-formation assay	Histology (HE-staining, immunofluorescence, TUNEL) ECG (left ventricular ejection fraction, fractional shortening, end-diastolic and end-systolic volumes)	Huang et al. (Ref. 45)
MTT assay FACS (Annexin V/PI) Western blot (bcl-2, activated caspase-3, autophagy-related protein p62, autophagy-associated protein LC3II/LC3I, FGFR1, PI3K/Akt pathway protein expression)	TTC staining (evidence for ischaemic/reperfusion injury) Western blot (bcl-2, autophagy-related p62, activated caspase-3, autophagy-associated protein LC3II/LC3I, PI3K/Akt pathway protein expression)	Ye et al. (Ref. 32)
Western blot (Bcl-xl, Bax, Nox2) FACS (Annexin V/PI) Oxidative damage indicators (DHE-ROS kit) Exosome uptake (fluorescence microscopy) RNA-Seq for differential expression of lncRNAs PDGFD expression (qPCR and Western blot)	EvansBlue/TTC staining (evidence for ischaemic/reperfusion injury) ECG, left ventricular systolic pressure, left ventricular developed pressure, maximal ventricular pressure rise during systolic period, maximal ventricular pressure decrease during diastolic period via cannulation insert MDA and SOD secretion levels (method not stated) PDGF expression (Western blot)	Zhai et al. (Ref. 39)
Cell counting kit-8 (viability) FACS (annexin-V/PI) Modified BMSCs (expressing miR-21-5p) Fluo-labelled qPCR (circRNA_0031672, PDCD4, miR-21-5p) Western blot (β-actin, PDC4)	Blood pressure ECG qPCR: myocardial tissue (circRNA_0031672, PDCD4, miR-21-5p) Western blot (β-actin. PDCD4)	Zhang et al. (Ref. 40)

*Abbreviations:* Akt: protein kinase B; Bax: Bcl-2-associated X protein; Bcl-2: B-cell lymphoma 2; Bcl-xl: B-cell lymphoma-extra-large; BrdU: Bromodeoxyuridine; CMs: cardiomyocytes; Connexin-43: Gap junction protein alpha 1; DAPI: 4′,6-diamidino-2-phenylindole; DHE: Dihydroethidium; ECG: electrocardiogram; EdU: 5-ethynyl-2′-deoxyuridine; FACS: fluorescence-activated cell sorting; FGFR1: fibroblast growth factor receptor 1; HE staining: haematoxylin and eosin staining; HO-1: heme oxygenase-1; LC3: microtubule-associated protein 1A/1B-light chain 3; MDA: malondialdehyde; MSCs: mesenchymal stem cells; MTT: 3-(4,5-dimethylthiazol-2-yl)-2,5-diphenyl tetrazolium bromide; Nox2: NADPH oxidase 2; Oct-4: octamer-binding transcription factor 4; P62/SQSTM1: Sequestosome 1; PDC4/PDCD4: programmed cell death protein 4; PDGFD: platelet-derived growth factor D; PDGFRb: platelet-derived growth factor receptor β; PGC1a: peroxisome proliferator-activated receptor gamma coactivator 1-alpha; PI: propidium iodide; RNA-Seq: RNA sequencing; ROS: reactive oxygen species; SOD: superoxide dismutase; Sox-2: SRY-box transcription factor 2; TTC: 2,3,5-triphenyltetrazolium chloride; TUNEL: terminal deoxynucleotidyl transferase dUTP nick end labelling; WB/Western blot: Western blotting; lncRNA: long non-coding RNA; mTFA: mitochondrial transcription factor A; miR-21-5p: MicroRNA-21-5p; miRNA: MicroRNA; qPCR/RT-qPCR: quantitative real-time PCR; α-SA: alpha-sarcomeric actinin; α-SMA: alpha-smooth muscle actin.

All authors reported that their respective *in vitro* model adequately recapitulated the beneficial effects observed *in vivo*. However, due to general model differences, comparisons were qualitative (e.g., the same trends towards myocardial repair were observed) rather than quantitative.

## Comparison of *in vitro* findings with literature data and translational aspects

Instead, or in addition to animal models, several authors used existing literature to correlate their experimental findings with known clinical data and outcomes. Yue et al. validated the influence of hypoxic stress on gene expression in iECs by comparing their *in vitro* generated results with animal *in vivo* and clinical datasets. This bioinformatic comparison revealed that the five most affected genes identified in the *in vitro* model show similar trends regarding up- and downregulation in rat ventricular tissue and in endothelial cells isolated from MI patients (Ref. 35). Hafez et al. emphasized the critical role of mitochondria in cardiac ischaemia by referencing earlier *in vivo* studies, aiming to confirm their findings *in vitro* (Ref. 20). Heinemann et al. supported their findings by citing data from literature that implicates endocannabinoids and the CB2 receptor in regulating inflammation and cardioprotection (Ref. 29), as demonstrated in rat hearts *ex vivo*. Chen et al. referred to studies demonstrating that genetic engineering of MSCs before transplantation enhances therapeutic effects, such as reducing infarct size, preventing cardiac remodelling and improving cardiac function in infarcted hearts (Ref. 36). Luo et al. compared their findings with results from the DANAMI-3-iPOST trial, which showed that ischaemic post-conditioning failed to replicate findings from rat models. Consequently, they initiated *in vitro* studies to explore the underlying mechanisms, explaining their use of rat cells (Ref. 27). Yang et al. observed that the extent of cardiomyocyte injury in their experiments did not align with the more severe ischaemic damage reported in *in vivo* studies, prompting further investigation (Ref. 22). Huang et al. linked their findings to human studies by comparing them with clinical trials involving MSC transplantation in patients (Ref. 45). Li et al. focussed on propofol, a commonly used anaesthetic in clinical practice, and explored its protective effects on myocardial I/R injury, as observed in clinical settings (Ref. 25).

Table 3 provides a structured overview of biomarkers used to validate *in vitro* myocardial ischaemia/reperfusion models, highlighting the predominance of nonspecific stress and viability markers and the limited use of clinically translatable endpoints.

**Table 3.** Biomarkers used for validation of *in vitro* myocardial ischaemia/reperfusion models, categorized by their primary application Table 3. long description.

A) Stress response/injury markers			
Biomarker/assay	Biological process	Measurement type	Limitations
Cell viability (live/dead, Calcein AM)	Cell survival	Direct staining	Highly nonspecific
Annexin V/PI	Apoptosis/necrosis	Fluorescence	No functional insight
Caspase-3/7 activity	Apoptosis	Enzymatic	Late-stage marker
ROS indicator dyes (DCF, DHE)	Oxidative stress	Fluorescence	Probe-dependent artefacts
HIF-1α	Hypoxic signalling	Protein expression	Indirect hypoxia readout
LDH release	Membrane integrity	Colorimetric	Not specific to oxidative stress
MTT/XTT	Metabolic activity	Colorimetric	Influenced by multiple pathways

B) Direct comparative *in vivo* validation markers			
Biomarker/outcome	*In vivo* correlate	Species	Interpretation
Infarct size	TTC staining	Mouse/rat	Structural myocardial damage
Fibrosis markers (collagen I/III)	Histology	Mouse/rat	Remodelling
Cardiac function (EF, FS)	Echocardiography	Mouse/rat	Functional recovery
Angiogenesis markers (VEGF, CD31)	Vessel density	Mouse/rat	Reparative response
Connexin-43	Electrical coupling	Mouse/rat	Cardiomyocyte integration

C) Clinical/preclinical reference markers		
Biomarker	Clinical relevance	Translational potential
Troponin T (TropT)	Gold standard MI biomarker	High - direct patient comparability
D-dimer	Thrombotic activity	Moderate–high
BNP/NT-proBNP	Cardiac stress	Moderate
Mitochondrial dysfunction markers	Energy failure	Indirect clinical relevance
Inflammatory cytokines (IL-6, TNF-α)	Systemic inflammation	Context-dependent

*Note:* Stress response and injury markers (A) assess cellular viability, apoptosis and oxidative stress; direct comparative *in vivo* validation markers (B) provide correlation with structural and functional myocardial outcomes in animal models; and clinical/preclinical reference markers (C) include biomarkers with translational relevance, facilitating comparison of *in vitro* findings with human myocardial infarction. References are provided for each marker as reported in the included studies.

### Limitations of *in vitro* MI co-culture models

We aimed to gain deeper understanding of the limitations in generating cardiac I/R co-culture models by identifying key factors that hinder their wider adoption and acceptance. To this end, we systematically analysed the constraints and challenges reported by respective study authors. Interestingly, a subset of studies did not explicitly mention any limitations, except for the general understanding that *in vitro* models are never a full representation of the *in vivo* situation and that animal testing would be required for verification of their data (Refs 16, 18, 20, 24, 26, 27, 36–40, 44).

#### Technical limitations

Several studies highlight technical difficulties with experimental systems, as it is not feasible to test all possible time points, oxygen gradients, media compositions and timing for treatments and cell therapy regiments to identify an ideal protocol (Refs 19, 22). Common concerns also include the brief exposure of cells to atmospheric oxygen concentrations after ischaemia modelling while preparing cells for analysis, which may induce oxidative stress responses – similar to reperfusion injury – and could affect results (Ref. 28). The well-controlled but artificial set up of *in vitro* tests might also hinder direct translation to the clinical setting: Li et al. identified optimal distances between cells co-cultured in transwells to see paracrine effects. However, the spacing and cell arrangement do not reflect the actual cell spacing *in vivo* (Ref. 23).

Additionally, technical issues arise with more complex culture formats: Mosadegh et al. pointed out that measuring CM contractility in their 3D-paper-based stacked model requires the system to be disassembled for analysis, potentially altering the metabolic state of cells and invalidating downstream testing results (Ref. 34). Another identified gap in the *in vitro* inflammatory pathway analysis is the predominant focus on single molecules guided by a priori knowledge or hypotheses, potentially overlooking broader processes and unknown effects (Ref. 45).

#### Cell-related limitations

While all studies aimed to include the most relevant cell types for their particular research questions, several authors reported the limited number of cell types – often only two – as a limiting factor (Refs 22, 23). The selection of cell types and cell sources was reported as another challenging factor: Primary human cells, while preferred for their physiological relevance, are difficult to obtain and maintain, leading to the use of animal cells and cell lines instead (Refs 17, 21, 25, 32, 34, 38). The use of primary material often results in low numbers of biological and technical replicates. Firouzi et al. and Yang et al. reported this limitation to reduce the robustness of their findings (Refs 15, 22). While iPSC-derived CMs are a common alternative to primary cardiomyocytes, they are inherently immature and therefore lack key characteristics of their mature *in vivo* counterparts, limiting the applicability of findings (Ref. 30).

## Discussion

The 28 publications analysed in this review addressed various aspects of MI and employed diverse cellular and technological approaches. The main similarities between studies were as follows: (1) the basic approach of mimicking I/R injury, for example, via hypoxia and serum and/or glucose starvation, (2) similar limitations regarding the restricted complexity of *in vitro* models, the use of immature CMs and (3) limited model verification.

### Biological aspects

#### Mixing of species and transferability to human biology

While we evaluated and compared the self-reported limitations of the different studies, we also aimed to identify general concerns regarding *in vitro* co-culture models of MI that have not been explicitly addressed in the reviewed articles. One largely unaddressed aspect is the combination of cellular species. Many studies combined cells from different species without discussing potential effects on generated data or the potentially reduced clinical relevance. Despite the common practice of mixing species for *in vitro* experiments, literature on this topic is sparse. Apart from mixing of species, many *in vitro* models rely entirely on cells from a single non-human species, potentially limiting the transferability to humans. Nevertheless, some authors compare their preliminary data generated using animal cells with their human cell-based *in vitro* systems. A notable example is the analysed publication by Firouzi et al., who progressed from their previously published murine ‘cardiochimera’ cells to human ‘cardiochimeras’ and compared their characterization data with their earlier findings (Ref. 15).

Three of the reviewed papers reported similar results while incorporating different cell types and species, indicating that for certain applications, species choices may be less crucial: Mahrouf-Yorgov et al. and Hafez et al. investigated mitochondrial transfer from MSCs in human cells (Refs 20, 33), while Han et al. conducted experiments using rat cells (Ref. 26). Despite these differences, all studies consistently demonstrated mitochondrial transfer and its protective effects. This highlights that animal-based models can capture clinically relevant aspects but should be validated against human data.

#### (Im)Maturity of cardiomyocytes

Multiple studies analysed the protective effects of different cell types on CMs using neonatal rat CMs or H9C2 cells (Refs 15, 17, 18, 19, 22, 25, 16, 27, 31, 32, 35–39, 44, 45). H9C2 cells, a cell line originating from embryonic BDIX rat heart cells (Ref. 48), are often used undifferentiated, as represented by the publication list analysed in this review (Refs 17, 18, 22, 25, 26, 27, 32, 36, 38, 40, 45). However, a study by Onódi et al. concluded that even in their differentiated state, H9C2 cells do not express a cardiac phenotype comparable to adult rat primary CMs. Onódi et al. additionally incorporated neonatal rat CMs in their experiments, which were found to resemble adult cardiac tissue more closely than differentiated H9C2 cells in terms of cardiac mRNA expression (Ref. 49). Nevertheless, both rat cell types represent partially immature CMs and do not reflect the human *in vivo* situation.

According to Onódi et al., hiPSC-CMs displayed higher cardiac mRNA expression than their differentiated human cell line counterpart and exhibited an adequate responsiveness to I/R stress (Ref. 49). Despite known limitations in maturation (Ref. 50), hiPSC-CMs may represent a more suitable model than animal-derived CMs. Given the limited availability of primary human cardiac cells, hiPSC-CMs could serve as a key alternative. This is particularly important for clinical translation, as findings from animal-based models should be re-evaluated and validated in human cell-based *in vitro* systems prior to progression to *in vivo* studies.

### Technical aspects

#### Oxygen levels

Among the analysed *in vitro* models of I/R injury, hypoxia was mainly utilized to simulate ischaemic conditions, with oxygen concentrations ranging from 0% to 3% O_2_, though few studies justify their chosen levels. This raises concerns about the physiological relevance of these models, as healthy tissues *in vivo* typically experience oxygen levels between 2% and 6% (physioxia) (Ref. 51). Studies suggest 5% to be the ideal physioxia level for myocardial tissues (Ref. 52), differing vastly from the atmospheric oxygen (18%–21%) commonly used in cell culture for reperfusion simulation (Refs 20–23, 26, 27, 28, 30, 31, 36). During MI, oxygen supply to cardiac tissue drops significantly from this physiological range, but exact values remain inconsistent due to biological variability and the severity of ischaemia (Ref. 53). Additionally, while reperfusion restores oxygen, sudden increases often exacerbate injury via ROS production (Ref. 54). Pre-clinical guidelines, therefore, advise against oxygen supplementation during MI, as it can elevate oxygen levels above normoxia and worsen reperfusion injury (Ref. 55). Consequently, modelling reperfusion injury *in vitro* with ‘normoxia’ levels of 18% to 21% O_2_ may distort the clinical relevance of findings. Moreover, the oxygen concentration in the culture medium surrounding cells *in vitro* does not necessarily equate to the oxygen levels experienced by cells within tissues *in vivo*. Factors such as oxygen diffusion gradients, cellular consumption rates and the three-dimensional architecture of tissues influence the actual oxygen tension that cells experience (Refs 56, 57).

Regarding the 28 studies analysed in this review, 2D transwell and ratio-based co-cultures were predominantly used, which are unable to mimic oxygen gradients. Transwell assays can be a useful tool for studying paracrine signalling, which was the main focus of five studies (Refs 17, 23, 28, 35, 37). Notably, one of those publications, namely, Yue et al., addressed paracrine signalling using a 3D approach without permitting cell–cell contacts (Ref. 35).

3D cell cultures are widely recognized for generating oxygen gradients with oxygen levels dropping towards the culture centre (Refs 58–61), making them a promising tool for MI-induced hypoxia. Yue et al. employed this approach (Ref. 35); however, the absence of oxygen measurements raises concerns regarding the scientific accuracy of their model.

Besides hypoxia, changing the media composition to model ischaemia further increases model complexity. A general consensus in the reviewed studies was the use of serum and glucose deprivation, either separately or simultaneously, with the latter being the preferred method. In four studies, other types of specialized media were used during the ischaemia phase (Refs 16, 17, 28, 29). While serum and/or glucose-deprived DMEM was the most abundantly used ischaemic media, there is insufficient data to conclude if this is truly the best option or rather had practical reasons.

### Limitations of this review

While this review uses methods from systematic reviews to decrease author bias, we are aware that it does not fully qualify as a systematic review. Particularly, data analysis is not based on statistical methods but describes and compares aspects of interest identified based on the authors’ expertise in the field. We found that numerical data in the reviewed set of publications was too inhomogeneous for any meaningful meta-analysis. Mainly, the articles reported different parameters, and even in cases where similar parameters were addressed, the underlying experimental methods were too fundamentally different for statistical comparisons.

Additionally, the list of publications included in this review might not fully cover the range of relevant literature. In the interest of objectivity and reproducibility, we only included publications found with our stated search query. We noticed that at least four relevant publications were missed by our query (Refs 62–65). In part, this disparity may be due to our inclusion criterion ‘co-culture’ (and alternative spellings). Hence, original articles not using the term throughout their text were excluded, even if they used cultures containing more than one cell type. Additionally, the wide range of currently available cardiac tissue models (Refs 66, 67, 68) suggests that other publications might have also fit within the scope of this review, but they were not found in our literature search.

### Future perspectives

Several of the reviewed studies employed non-human cells or combined cells from different species to generate *in vitro* MI co-culture models. To date, no studies have systematically examined the potential impact of such mixed cultures on experimental outcomes or clinical relevance. Future investigations should specifically benchmark animal-based, inter-species and human *in vitro* models against each other, as well as against *in vivo* animal data and clinical observations. Establishing a consensus on standard experimental parameters, including typical ischaemia and reperfusion durations as well as core readouts, will be critical to allow meaningful comparisons between emerging models. Defining such benchmarks, as further discussed in the Expert Topical Summary, represents a key step towards harmonizing experimental designs and improving translational relevance.

Looking forward, patient-derived iPSCs offer a promising avenue for developing personalized MI models. These cells provide the opportunity to capture patient-specific genetic and functional variability, enabling more predictive and clinically relevant *in vitro* systems. Coupling iPSC-derived cardiomyocytes, endothelial cells and supporting cell types in physiologically relevant co-cultures could substantially improve the translational value of *in vitro* findings.

Advances in microfluidic and organ-on-chip platforms further enhance the potential of *in vitro* MI modelling. These systems allow for dynamic perfusion, controlled mechanical loading and real-time monitoring of cellular responses, providing a closer approximation of the *in vivo* myocardial environment. Integrating human cell sources with organ-on-chip technologies could, therefore, bridge critical gaps between conventional cell culture and clinical pathophysiology. In the longer term, omics-based approaches (e.g., transcriptomics, proteomics) may also warrant evaluation to determine whether they can provide robust and comprehensive validation strategies for these complex *in vitro* models. The development and rigorous validation of complex, physiologically relevant *in vitro* models – including appropriate oxygen levels, ischaemia/reperfusion durations, three-dimensional tissue architecture, patient-specific cell sources and standardized readouts – will be essential for improving reproducibility, clinical translatability and the overall impact of MI research.

## Expert and topical summary

This review highlights a substantial lack of standardization in *in vitro* models of ischaemia/reperfusion (I/R) injury. Experimental approaches remain highly heterogeneous, with a continued predominance of immortalized, frequently non-human, cell lines despite well-recognized limitations in their clinical translatability. While *in vivo* models and bioinformatic approaches are often employed to bridge the translational gap, the intrinsic limitations of *in vitro* systems are rarely addressed in a systematic manner. When discussed, concerns most commonly relate to the use of non-human or immortalized cells and the challenges of accurately reproducing hypoxic conditions *in vitro*. Notably, hypoxia is frequently operationally defined as 0% oxygen and normoxia as approximately 20% oxygen, largely neglecting the concept of physiological hypoxia characteristic of myocardial tissue.

Within this context, several conceptual gaps remain unresolved. Based on the available literature, it is not possible to clearly identify the specific barriers preventing the establishment of a standardized *in vitro* I/R protocol. Despite the relatively conserved pathophysiology of I/R injury across experimental systems, the extensive variability in model design, cell sources, oxygen tensions and experimental endpoints precludes robust conclusions regarding the underlying causes of this lack of consensus.

Questions concerning the most appropriate cell types, hypoxic culture conditions and molecular or functional readouts that best recapitulate human I/R injury therefore arise frequently. However, even when examined in greater detail, the existing evidence remains highly heterogeneous. No clearly defined best practice emerges with respect to the use of primary human cells versus commonly employed cell lines, the definition of ischaemic and reperfusion phases, or the selection of outcome measures with demonstrable clinical relevance. Similarly, there is a paucity of systematically validated *in vitro* markers that reliably correlate with clinical outcomes, limiting meaningful extrapolation from bench to bedside.

The authors note a general trend towards the use of primary human cell material in an effort to enhance the reliability and clinical relevance of *in vitro* findings. However, this approach inherently introduces biological variability between donor samples, which can lead to increased experimental variability. In addition, the use of primary human cells is often costly, technically demanding, and labour intensive, factors that likely contribute to the continued widespread reliance on immortalized cell lines, frequently derived from non-human species. We hypothesize that these constraints are driven predominantly by considerations of time, cost and logistical feasibility rather than by a deliberate prioritization of clinical relevance. Nevertheless, until compelling evidence suggests otherwise, primary human cells should be regarded as the most physiologically relevant *in vitro* cell source for modelling human myocardial injury.

Across the studies reviewed, both hypoxia and reperfusion durations varied substantially, with a frequent tendency towards prolonged exposure times of approximately 24 h for each phase. When contrasted with clinical data on symptom-to-treatment intervals, this represents a clear discrepancy. In clinical settings, total ischaemic time is most commonly reported to range between approximately 3.5 and 5 h (Refs 69–72), with European Society of Cardiology guidelines targeting a window of no more than 2 h from electrocardiographic diagnosis to reperfusion therapy (Ref. 41). Harmonization of *in vitro* myocardial infarction models should therefore aim to better align experimental ischaemia and reperfusion durations with these clinically relevant timeframes in order to more accurately reflect real-life ischaemic injury.

To further strengthen translational relevance, the authors suggest that future studies incorporate clinically meaningful endpoints as previously identified in this review. For example, levels of established biomarkers such as cardiac troponin T (TropT) measured in patients undergoing myocardial infarction and subsequent coronary angiography appear highly comparable and could be directly correlated with corresponding concentrations detected in cell culture supernatants, providing a potential means to assess cardiomyocyte injury *in vitro* in a clinically interpretable manner. The selection and validation of additional biomarkers with direct clinical comparability should ideally be undertaken in close collaboration with clinical experts.

Advancing the field will, therefore, require coordinated and collaborative efforts aimed at harmonizing experimental designs. Multi-centre comparative studies, systematic evaluations of primary human cells versus commonly used cell lines and rigorous validation of clinically relevant biomarkers will be essential to improve reproducibility, enhance translational value and ultimately establish robust, human-relevant *in vitro* alternatives to animal models in ischaemia/reperfusion research.

## Supporting information

10.1017/erm.2026.10058.sm001 Lugmayr et al. supplementary material 1 Lugmayr et al. supplementary material

10.1017/erm.2026.10058.sm002 Lugmayr et al. supplementary material 2 Lugmayr et al. supplementary material

## Supplementary material

The supplementary material for this article can be found at http://doi.org/10.1017/erm.2026.10058.

## Long descriptions

### Figure 1. Long description

The flowchart consists of five main vertical stages in pink boxes and three exclusion branches in light pink boxes to the right.

1. Identification: Records identified through database search n equals 270.

2. Screening: Records screened for topic relevance Myocardial infarction n equals 270. An arrow points right to Records excluded: Not myocardial infarction as main topic n equals 208.

3. Eligibility: Full-text articles assessed for eligibility M I studies n equals 62. An arrow points right to Full-text articles excluded: No in vitro M I model n equals 27; Excluded due to study focus and cell types n equals 2; No co-culture experiments n equals 4. Total excluded n equals 33.

4. Inclusion Criteria: Studies meeting inclusion criteria M I in vitro co-culture studies n equals 29. An arrow points right to Studies excluded after detailed evaluation: Insufficient method description n equals 1.

5. Final Synthesis: Studies included in qualitative synthesis / review n equals 28.

Navigate back to Figure 1..

### Figure 3. Long description

The figure consists of two vertically stacked bar charts labeled a and b.

Panel a, titled Hypoxic Stress Duration, features an orange bar chart. The y-axis is labeled Number of Publications and ranges from 0 to 8. The x-axis is labeled Duration [Hours] and lists discrete time points. The data shows:

* 1h: 2 publications

* 2h: 2 publications

* 3h: 1 publication

* 4h: 3 publications

* 5h: 1 publication

* 6h: 4 publications

* 9h: 1 publication

* 12h: 4 publications

* 24h: 7 publications (the peak)

* 48h: 2 publications

* 72h: 2 publications

* 168h: 1 publication

Panel b, titled Reoxygenation Duration, features a dark red bar chart. The y-axis is labeled Number of Publications and ranges from 0 to 6. The x-axis is labeled Duration [Hours]. The data shows:

* 0.5h: 1 publication

* 1h: 1 publication

* 2h: 2 publications

* 3h: 3 publications

* 4h: 3 publications

* 6h: 3 publications

* 8h: 2 publications

* 9h: 1 publication

* 12h: 1 publication

* 15h: 1 publication

* 16h: 3 publications

* 17h: 1 publication

* 24h: 5 publications (the peak)

* 48h: 1 publication

* 72h: 1 publication

Navigate back to Figure 3..

### Table 1. Long description

The table consists of two columns: Condition and Media composition.

* Glucose deprivation: Includes 0 percent glucose D M E M and glucose deprivation before hypoxia.

* Serum deprivation: Includes serum-free medium before co-culturing with stem cells, overnight serum-starvation, and serum-free E G M-2 medium.

* Combined approaches (glucose and serum deprivation): Includes glucose- and serum-free D M E M-F 12, serum-free and glucose-free D M E M, serum-free D M E M with low (1000 mg/L) glucose, and unspecified glucose- and serum-free culture medium.

* Specialized buffers/media: Includes ischaemic mimetic solution with precise ionic concentrations, 2-deoxyglucose and sodium dithionite solution, I M D M with beta-mercaptoethanol and interferon-gamma, M 199 with penicillin-streptomycin, and D M E M with 10 percent serum.

* Other I/R inducing agents and treatments: Includes C o C l sub 2-containing medium, 600 micromolar H sub 2 O sub 2, 1 micromolar Doxorubicin, paraffin overlay, and overlay with fibroblast layers.

Navigate back to Table 1..

### Figure 4. Long description

Panel a, titled Species at the center, shows a radial diagram divided into six segments. Starting from the top-left and moving clockwise: Human accounts for 7 publications (maroon), Rat for 7 (dark purple), Mouse for 2 (deep violet), Human-Rat for 6 (yellow), Mouse-Rat for 4 (pale orange), and Swine-Rat for 1 (bright orange).

Panel b, titled Source at the center, shows a radial diagram divided into three segments. Starting from the top-right and moving clockwise: Primary Cells account for 11 publications (maroon), Cell Lines for 4 (bright orange), and Combined for 12 (pale orange).

Navigate back to Figure 4..

### Figure 5. Long description

Three donut charts categorized by species.

Top-left chart, Rat. Starting from the top clockwise.

- H 9 C 2, 11 publications, orange.

- Neonatal Cardiomyocytes, 7 publications, dark purple.

- Fibroblasts, 4 publications, medium purple.

- Mesenchymal Stem Cells, 3 publications, dark purple.

- Polymorphonuclear Cells, 1 publication, light purple.

- Vascular Smooth Muscle Cells, 1 publication, dark purple.

- R B L-2 H 3, 1 publication, orange.

Top-right chart, Mouse. Starting from the top clockwise.

- Embryonic Cardiomyocytes, 1 publication, dark purple.

- Adult Cardiomyocytes, 3 publications, dark purple.

- Endothelial Cells, 1 publication, medium purple.

- Cardiac Progenitor Cells, 1 publication, dark purple.

- Macrophages, 2 publications, light purple.

- 3 T 3 Cells, 1 publication, orange.

Bottom chart, Human. Starting from the top clockwise.

- E A dot hy 9 2 6, 1 publication, orange.

- T H P-1, 1 publication, orange.

- Cardiomyocytes, 2 publications, dark purple.

- Cardiac Interstitial Cells, 1 publication, medium purple.

- Cardiac Progenitor Cells, 1 publication, dark purple.

- Cardiac Stem Cells, 1 publication, medium purple.

- Mesenchymal Stem Cells, 5 publications, dark purple.

- Human Umbilical Vein Endothelial Cells, 3 publications, medium purple.

- Saphenous Vein-derived Pericytes, 1 publication, light purple.

- i P S C-derived Cardiomyocytes, 3 publications, light blue.

- i P S C-derived Fibroblasts, 1 publication, medium blue.

- i P S C-derived Endothelial Cells, 1 publication, dark blue.

- M Y L 2 Venus, 1 publication, yellow-orange.

- R L 1 4, 1 publication, orange.

Navigate back to Figure 5..

### Figure 6. Long description

The image contains two panels labeled a and b.

Panel a, titled Viability and Apoptosis Assessment, is a donut chart with four segments in shades of purple. Starting from the top-left and moving clockwise:

* T U N E L Assay: 5

* Caspase Assay: 7

* C C K-8 Assay: 3

* Viability Stains: 17

Panel b, titled Oxidative Stress Assessment Methods, is a donut chart with seven segments in shades of orange and brown. Starting from the top-left and moving clockwise:

* A T P Dye: 2

* R O S Dye: 2

* O C R Measurement: 2

* H I F 1 alpha Measurement: 1

* L D H Assay: 3

* M T T Assay: 5

* Gene Expression (R N A seq): 7

* M M P Assay: 1

Navigate back to Figure 6..

### Table 2. Long description

The table consists of three columns: in vitro markers, in vivo markers, and Authors.

* Row 1: in vitro markers include R T - q P C R for cardiac markers, H sub 2 O sub 2-induced R O S and apoptosis assay, Western blot for pro-inflammatory cytokines, and T U N E L assay. in vivo markers include Histology and immunofluorescent labelling of infiltrating macrophages. Authors: Aonuma et al.

* Row 2: in vitro markers include Immunofluorescence microscopy, F A C S, cell adhesion, dye transfer, and Western blot. in vivo markers include Histology combined with immunofluorescence microscopy for engraftment assessment. Authors: Popara et al.

* Row 3: in vitro markers include F A C S, oxygen consumption rate, T E M, heme oxygenase activity, immunocytochemistry, and R T - q P C R. in vivo markers include Troponin T in mouse serum, Western blot for Caspase-3, and R T - q P C R. Authors: Mahrouf-Yorgov et al.

* Row 4: in vitro markers include F A C S, immunofluorescence for various factors like Oct-4 and Sox-2, R T - q P C R, and several assays (E d U, T U N E L, Caspase 3/7, Br d U, migration, Matrigel). in vivo markers include E C G, hemodynamic measurements, immunohistochemistry, T U N E L assay, and infarct size assessment. Authors: Avolio et al.

* Row 5: in vitro markers include time-lapse imaging and calcium transients. in vivo markers include immunostaining, E C G, and infarct size analysis. Authors: Wang et al.

* Row 6: in vitro markers include F A C S and Matrigel-tube-formation assay. in vivo markers include histology and E C G. Authors: Huang et al.

* Row 7: in vitro markers include M T T assay, F A C S, and Western blot for Bcl-2 and autophagy proteins. in vivo markers include T T C staining and Western blot for similar proteins. Authors: Ye et al.

* Row 8: in vitro markers include Western blot, F A C S, oxidative damage indicators, exosome uptake, R N A - Seq, and P D G F D expression. in vivo markers include Evans Blue/T T C staining, E C G, M D A and S O D secretion, and P D G F expression. Authors: Zhai et al.

* Row 9: in vitro markers include Cell counting kit-8, F A C S, modified B M S C s, q P C R, and Western blot. in vivo markers include blood pressure, E C G, q P C R, and Western blot. Authors: Zhang et al.

Navigate back to Table 2..

### Table 3. Long description

The table is divided into three sections.

Section a: Stress response or injury markers.

- Cell viability (live/dead, Calcein A M): Biological process is Cell survival; Measurement type is Direct staining; Limitations are Highly nonspecific.

- Annexin V / P I: Biological process is Apoptosis or necrosis; Measurement type is Fluorescence; Limitations are No functional insight.

- Caspase-3/7 activity: Biological process is Apoptosis; Measurement type is Enzymatic; Limitations are Late-stage marker.

- R O S indicator dyes (D C F, D H E): Biological process is Oxidative stress; Measurement type is Fluorescence; Limitations are Probe-dependent artefacts.

- H I F-1 alpha: Biological process is Hypoxic signalling; Measurement type is Protein expression; Limitations are Indirect hypoxia readout.

- L D H release: Biological process is Membrane integrity; Measurement type is Colorimetric; Limitations are Not specific to oxidative stress.

- M T T / X T T: Biological process is Metabolic activity; Measurement type is Colorimetric; Limitations are Influenced by multiple pathways.

Section b: Direct comparative in vivo validation markers.

- Infarct size: in vivo correlate is T T C staining; Species is Mouse or rat; Interpretation is Structural myocardial damage.

- Fibrosis markers (collagen I / III): in vivo correlate is Histology; Species is Mouse or rat; Interpretation is Remodelling.

- Cardiac function (E F, F S): in vivo correlate is Echocardiography; Species is Mouse or rat; Interpretation is Functional recovery.

- Angiogenesis markers (V E G F, C D 31): in vivo correlate is Vessel density; Species is Mouse or rat; Interpretation is Reparative response.

- Connexin-43: in vivo correlate is Electrical coupling; Species is Mouse or rat; Interpretation is Cardiomyocyte integration.

Section c: Clinical or preclinical reference markers.

- Troponin T (Trop T): Clinical relevance is Gold standard M I biomarker; Translational potential is High - direct patient comparability.

- D-dimer: Clinical relevance is Thrombotic activity; Translational potential is Moderate to high.

- B N P / N T-pro B N P: Clinical relevance is Cardiac stress; Translational potential is Moderate.

- Mitochondrial dysfunction markers: Clinical relevance is Energy failure; Translational potential is Indirect clinical relevance.

- Inflammatory cytokines (I L-6, T N F-alpha): Clinical relevance is Systemic inflammation; Translational potential is Context-dependent.

Navigate back to Table 3..
