# Supplementary material for: Current in vitro Myocardial Infarction Co-Culture Models and their Verification for Clinical Relevance
Source: Expert Rev Mol Med. 2026 Jun 15;28:e41. doi: 10.1017/erm.2026.10058 (PMC13761295; doi:10.1017/erm.2026.10058)
Supplement: Supplementary file 1 [file S1462399426100581sup002.docx]

**Supplementary Material**

**Current *in vitro* myocardial infarction co-culture models and their verification for clinical relevance**

Sara Lugmayr^1,2,#^, Elisabeth Heuböck^1,2,#^, David Bernhard^1,2^, Markus Mandl^1,2,+^ , Verena Charwat^1,2,+,^*

#/+ These authors contributed equally

^1^ Institute for Physiology and Pathophysiology, Department of Pathophysiology, Johannes Kepler University Linz, Krankenhausstrasse 5, 4020 Linz and Altenberger Strasse 69, 4040 Linz, Austria

^2^ Clinical Research Institute for Cardiovascular and Metabolic Diseases, Medical Faculty, Johannes Kepler University Linz, Altenberger Strasse 69, 4040 Linz, Austria

*Corresponding author:

DI^in^ (FH) Verena Charwat, PhD

Institute for Physiology and Pathophysiology, Department of Pathophysiology,

Johannes Kepler University Linz, Krankenhausstrasse 5, 4020 Linz

ORCiD:	<https://orcid.org/0000-0002-2222-3702>

Mail:	[verena.charwat@jku.at](mailto:verena.charwat@jku.at)

**Acknowledgements**

Supported by Johannes Kepler University Open Access Publishing Fund and the Federal State Upper Austria. The AI Tool ChatGPT versions 4.1 to 5.5 was used to refine the wording.

**Financial support**

M.M. and V.C. received financial support from the Medical Faculty through the internal funding program Impetus (I-06-24). This research was published with the support of the Open Access Publication Fund of the Johannes Kepler University Linz and the federal state Upper Austria.

**Conflict of interest**

Conflicts of Interest: Sara Lugmayr, Elisabeth Heuböck, David Bernhard, Markus Mandl and Verena Charwat declare none.

**Ethical standards**

This article is a review of previously published studies. The authors did not conduct any experiments involving human participants or animals. While the authors assert that all procedures described in the reviewed studies were reported as complying with the ethical standards of the relevant national and institutional committees on human experimentation and with the Helsinki Declaration of 1975, as revised in 2008, as well as with the appropriate guidelines for the care and use of laboratory animals, we cannot independently guarantee full compliance, as the included data are derived from external sources.

| **Ischemia/Reperfusion Stress Treatments** | | | | | | | | | |
| --- | --- | --- | --- | --- | --- | --- | --- | --- | --- |
| **#** | **Publlication title and first author** | **Ischemia** | | | | **Reperfusion** | | **Additional Information** | |
|  |  | **Stressor** | **Duration** | **Incubator/ Chamber** | **Medium (hypox)** | **Stressor** | **Duration** | **Optimum determined** | **Rationale for stress condition** |
| **1** | „Three-Dimensional Paper-Based Model for Cardiac Ischemia”, **Mosadegh** | S= 3-9 layers above CM (=2x CM layer, fibroblast layers above)  No control condition, only different number of layers | 7 days | Incubator  (37 °C and 5% CO_2_) | NA | NA | NA | No | - |
| **2** | “Transcriptome profiling of 3D co-cultured cardiomyocytes and endothelial cells under oxidative stress using a photocrosslinkable hydrogel system”,  **Yue** | NA | NA | NA | NA | S= 0.2 mM H_2_O_2_ (16h) followed by normoxic standard culture media (2h)  C= Normoxic media for 18 hours (MC at 16 hours)  Normoxia O_2_ percentage is not stated | 16 hours +  2 hours | No | - |
| **3** | “Development of a Cell Co-Culture Model to Mimic Cardiac Ischemia/Reperfusion In Vitro”, **Li** | S= 0.01% O_2_/ 5% CO_2,_ 37°C  C= normoxia (21% O_2_, 5% CO_2_, 74% N_2_, 37 °C) | 24 hours | Hypoxia chamber  (For stress treatment: flushed w/ 0.0125% O_2_, 5% CO_2_, 94.99%  N_2_) | 0% Glucose flushed w/ hypoxic gas (0.0125% O_2_, 5% CO_2_, 94.99% N_2_) | Two different conditions stated as S= 1) Normal culture conditions (21% O_2_, 5% CO_2_, 74% N_2_, 37 °C)  2) Figure states 95% O_2_, 5% CO_2_ as normal culture condition (seems excessive, probably 1) statement is true) | 2 hours | No | - |
| **4** | „Modelling myocardial ischemia/reperfusion injury with inflammatory response in human ventricular cardiac organoids“,  **Zhang** | S= 0.1% O_2,_ 5% CO_2,_ 37°C  C= 37°C,  5% CO_2_, 21% O_2_ | 24 hours | Incubator (37C, 5% CO_2_ and 0.1% O_2_) | DMEM-F12 w/o Glucose or serum | S= Normal culture conditions (37°C,  5% CO_2_ and 21% O_2_) | 24 hours | Yes, durations for hypoxia and reoxygenation | Proportions of surviving/early apoptosis and late apoptosis at 50:25:22, “ensuring adequate but not  excessive damage, consistent with the characteristics of I/R injury” |
| **5** | “Development of an In Vitro Cardiac Ischemic Model Using Primary Human Cardiomyocytes”,   **Hafez** | S= 1% O_2_  C= High glucose, phenol-free DMEM  + 10% FBS  in normoxic condition (21% O_2_ and  5% CO_2_) | 30 min | Incubator (1% O_2_, 5% CO_2_, 94% N_2_) | Serum-free, glucose-free and phenol-free (pre-incubated in hypoxic environment for 24 hours) | S= Normoxic condition  (21% O_2_ and  5% CO_2_) | 3 hours | Yes, for hypoxia (3 hours) and reoxygenation (6 hours) | **Hypox.:** HIF-1α expression; optimal duration = first time point whereby 100% of the cells are positive for HIF-1α  **Optimal hypoxic/reox.**: “based on 50% mitochondrial injury via 3-(4,5  dimethylthiazol-2-Yl)-2,5-diphenyltetrazolium bromide assay  and cytotoxicity via lactate dehydrogenase (LDH) assay”  **Reox.:** 6 hours was determined optimal to induce reversible cell injury |
|  |  |  | 1 hour |  |  |  |  |  |  |
|  |  |  | 2 hours |  |  |  | 6 hours |  |  |
|  |  |  | 3 hours |  |  |  |  |  |  |
|  |  |  | 6 hours |  |  |  | 9 hours |  |  |
|  |  |  | 12 hours |  |  |  |  |  |  |
| **6** | “Human CardioChimeras: Creation of a Novel “Next-Generation Cardiac Cell”,  **Firouzi** | - | - | Assumably standard incubator with 21% O_2_, but n.s. | **NRCM** **co-culture** **with stem cells**  S= NRCM: 24 hours serum-free medium; then stem cells were added (1:5 ratio) for 24 hours | NA | NA | Not in this paper, but group published a similar paper where they created murine cardiochimeras, which was the basis for this paper | - |
|  |  |  |  |  | C1= Medium with 10% FBS added instead of stem cells for rescue NRCM  C2= constant culture in medium with 10% FBS |  |  |  |  |
| **7** | “Combined Intramyocardial Delivery of Human Pericytes and Cardiac Stem Cells Additively Improves the Healing of Mouse Infarcted Hearts Through Stimulation of Vascular and Muscular Repair”,  **Avolio** | NA | 48 hours | Incubator | **CSC + SVP co-culture (direct and Transwell):**  S= EGM-2 serum-free medium  C= CSC/SVP mono-culture | NA | NA | No | - |
|  |  | **CSC + SVP mono- and direct co-culture:**  S= 2% O_2_, 5% CO_2_  C= normoxia (20% O_2_) | 48 hours | Incubator | Serum-free EGM-2 medium  (also used for control) | NA | NA | No | - |
| **8** | “Bone marrow‑derived mesenchymal stem cells rescue injured H9c2 cells via transferring intact mitochondria through tunneling nanotubes in an in vitro simulated ischemia/reperfusion model”,  **Han** | S= 95% N_2_, 5% CO_2_  C= 5% CO_2_ and 95% air at 37˚C | 12 hours | Hypoxic chamber | S= Serum‑ and glucose‑deficient DMEM  C= complete medium throughout experiment | S= DMEM 10% FBS; 5% CO_2_ and 95% air at 37˚C (Co-culture: BM-MSC added during reoxygenation) | 6 hours | No | - |
| **9** | “Apoptosis-Resistant Cardiac Progenitor Cells Modified With Apurinic/Apyrimidinic Endonuclease/Redox Factor 1 Gene Overexpression Regulate Cardiac Repair After Myocardial Infarction”,  **Aonuma** | **Co-culture NRVM + APE1-CPCs:** S= Anoxic environment | 3 days | AnaearoPack-Anaero | Serum-free medium | NA | NA | Yes, but only vaguely described  (see info in next column) | “After  NRVMs were at 50% confluence, APE1-CPCs were added at the  ratios of 15:1,6:1,3:1, and 2:1, in **varying concentrations of atmospheric oxygen**, to determine the necessary conditions **to best**  **visualize NRVM apoptosis**.” |
|  |  | **Co NRVM + APE1-CPCs (treated with APE1-antagonist):**  S= Anoxic environment | 2 days | AnaearoPack-Anaero | Serum-free medium | NA | NA |  |  |
| **10** | “Dedifferentiation, Proliferation and Redifferentiation of Adult Mammalian Cardiomyocytes after Ischemic Injury”,  **Wang** | (Experiment to study role of Connexin-43, using Cx43-knockdown ACMs + NRVMs) S= 5%O_2_, 5% CO_2_ (balance N_2_)  C= scramble siRNA-kd ACM | 72 hours | Environmental chamber | - | NA | NA | No | - |
| **11** | “Human cardiac progenitor cell activation and regeneration mechanisms: Exploring a novel myocardial ischemia/reperfusion in vitro model”,  **Sebastiano** | S= N_2_ gaseous environment  C= 3% O_2_  (“myocardial physiologic normoxia”) | 5 hours | Humidified incubator (5% CO_2_, 95% air) | Ischemic mimetic solution (in mM:  NaCl, 135; KCl, 8; MgCl_2_, 0.5;NaH_2_PO_4_, 0.33; HEPES,  5.0; CaCl_2_, 1.8; Na+-lactate, 20; pH= 6.8) | S= Normal culture conditions (3% O_2_) | Different analysis timepoints:  Before stress treatment,  post ischemia, and 1/3/ 8/16 hours  post reperfusion | No | - |
| **12** | “Mesenchymal stem cells sense mitochondria released from damaged cells as danger signals to activate their rescue properties”,  **Mahrouf-Yorgov** | Oxidative stress/ROS injury – hMADS, HUVEC or RL14 before co-culture:  S= 600 µM H_2_O_2_  C= no H_2_O_2_ treatment/ non-injured cells for co-culture | 2 hours | Incubator (5% CO_2_ atmosphere at 37 °C) | - | NA | NA | No | - |
|  |  | Damaging agent – hMADS, HUVEC or RL14 before co-culture:  S= 1 µM Doxorubicin  C= no Doxorubicin treatment/ non-injured cells for co-culture | 24 hours | Incubator (5% CO_2_ atmosphere at 37 °C) | - | NA | NA | No | - |
| **13** | “CB2 receptor-mediated effects of pro-inflammatory macrophages  influence survival of cardiomyocytes”  **Heinemann** | S= 2% O_2_, 5% CO_2_  C= 21% O_2_, 5%CO_2_ | 1 and 24h | CO_2_ incubator Heraeus BBD6220, Thermo Fisher Scientific | IMDM w/ P/S, 0,1% beta-mercaptoethanol and FCS + IFN-gamma 100ng/mL for 24h. | NA | Not specified “experiments were stopped by discarding medium and fixing cells with PFA” | No | - |
| **14** | “Silica Nanoparticles Actively Engage With Mesenchymal Stem Cells in Improving Acute Functional Cardiac Integration”  **Popara** | H9C2: S=150 min 0% O_2_ then 1% O_2_ 5% CO_2_ co culture  C= 21% O_2_, 5%CO_2_ | 24h “Hypoxia” | Hypoxia chamber | Glucose-free medium | NA | No reperfusion | No | - |
| **15** | “Co-expression of Akt1 and Wnt11 promotes the proliferation and cardiac differentiation of mesenchymal stem cells and attenuates hypoxia/ reoxygenation-induced cardiomyocyte apoptosis“  **Bangdang** | S= Chamber containing a mixture of gases (94% N_2_,5%CO_2_, and 1% O_2_)  C= 21% O_2_, 5%CO_2_ | for 6h | Hypoxia chamber | Not specified | S= 5% CO_2_, 95% “air” | 24h | No | - |
| **16** | “microRNA-423-3p exosomes derived From cardiac fibroblasts mediates the Cardioprotective effects of ischaemic post-conditioning”  **Luo** | S= 95%N_2_,  5%CO_2_,  O_2_ below 1%  C= 21% O_2_, 5%CO_2_ | 9h then 5 min break then again 5 mins hypoxia (3 times shifted between H/N) with 5 mins each) | Hypoxic incubator | Culture medium deprived of glucose and serum for Hypoxia, complete DMEM with glucose, FBS and P/S for normoxia | S= “normal air” | 15h | No | - |
| **17** | “Feasibility Analysis of Oxygen‑Glucose Deprivation‑Nutrition Resumption on H9c2 Cells In vitro Models of Myocardial Ischemia‑Reperfusion Injury”  **Yang** | S= Hypoxia 0.5% O_2_, 5% CO_2_, and 94.5%N_2_  C= 21% O_2_, 5%CO_2_ | 6h | Tri-gas incubator (Huaxi electricity, china) | Major difference of this paper were different media under treatment (SI = ischaemia, SIR = Ischaemia reperfusion)    | S= 5% CO_2_, 21% O_2_, and balance N_2_ at 37°C | 0,5h and 12h | Comparison of 6 models with different media compositions | Literature from Pubmed searching “myocardial reperfusion injury H9c2 cells” – 6 models recreated |
| **18** | “Endothelial-Cell-Derived Human Secretory Leukocyte Protease Inhibitor (SLPI) Protects Cardiomyocytes against Ischemia/Reperfusion Injury”  **Kongpol** | S= Ischaemia Buffer (see on right)  Hypoxia by overlaying sample with paraffin | Ischaemia 40 mins buffer  Paraffin 1h |  | simulated ischemic basic buffer (137 mM NaCl,  3.8 mM KCl, 0.49 mM MgCl_2_, 0.9 mM CaCl_2_, 4.0 mM HEPES) containing 20 mM 2-deoxyglucose,  20 mMs odiumlactate, and 1 mM sodium dithionite at pH 6.5 | S= Change to a “complete medium” | 24h after medium change from buffer  3h after hypoxia | No | - |
|  |  | C= placement in complete medium |  |  |  |  |  |  |  |
| **19** | “Atorvastatin enhances the therapeutic efficacy of mesenchymal stem cells-derived exosomes in acute myocardial infarction via up-regulating long non-coding RNA H19”  **Huang** | S= “Hypoxia/serum deprivation” (not specified)  C= not specified, assume normoxia and no serum starvation | 12h | n/s | n/s, serum deprived medium | NA | NA | No | - |
| **20** | “Comparative Effect of MSC Secretome to MSC Co-culture on Cardiomyocyte Gene Expression Under Hypoxic Conditions *in vitro“* **Kastner** | S= Hypoxia, O_2_ below 1%  C= 21% O_2_, 5%CO_2_ | 2h | Hypoxia chamber (Billups-Rothenberg) | M199 + 1% P/S | S= Normal oxygen (ambient air) | time periods after reoxygenation were 4, 8, 24, 48, and 72 h | No | - |
| **21** | “Hypoxia-reoxygenation induces macrophage polarization and causes the release of exosomal miR-29a to mediate cardiomyocyte pyroptosis”  **Wang** | S= Hypoxia, 94% N_2_,5%CO_2_, and 1% O_2_  C= 20% O_2_, 5%CO_2_ | 12h | Hypoxia incubator Galaxy® 48 R | DMEM complete medium with 10%FBS | S= Normal air oxygen concentration | 6h | No | - |
| **22** | “Dexmedetomidine attenuates myocardial ischemia-reperfusion injury in vitro by inhibiting NLRP3 Inflammasome activation” **Huang** | S= Hypoxia 1% O_2_/5% CO_2_/94% N_2_  C = 5% CO_2_, 95% air | 6h | Tri-Gas Incubator | Low Glucose Medium | S= “Normoxic conditions” | 4h | No | - |
| **23** | “Propofol pretreatment alleviates mast cell degranulation by inhibiting SOC to protect the myocardium from ischemia-reperfusion injury” **Li** | S= Hypoxia 95% N_2_, 5% CO_2_  C = 5% CO_2_, 95% air | 4h | n.s. | DMEM + 10% FBS (+Propofol) | S= 95% O_2_ (listed like this in paper, may be an error, seems excessive), 5% CO2 | 4h | No | - |
| **24** | “Follistatin-like 1 promotes proliferation of matured human hypoxic iPSC-cardiomyocytes and is secreted by cardiac fibroblasts” **Peters** | S= Hypoxia 1% O_2_ or <1%  C = n.s. | 4h /24h | BakerRuskinn InvivO2 1000/BD GasPak EZ Pouch system | RPMI 1640 w/o glucose | NA | NA | No | - |
| **25** | Vascular smooth muscle cells activate PI3K/Akt pathway to attenuate myocardial ischemia/reperfusion-induced apoptosis and autophagy by secreting bFGF **Ye** | NA | 6h | n.s. | CoCl_2_-containing medium | S=Normal medium | 16h | No | - |
| **26** | Mitochondrial Respiratory Capacity is Restored in Hibernating Cardiomyocytes  Following Co-Culture with Mesenchymal  Stem Cells **Hocum Stone** | S= Hypoxia 1% O2, 5% CO2  C= n.s. | 24h | Evos FL Auto Onstage Incubator (ThermoFisher, Waltham, MA, USA) | Not specified | S= Normoxic conditions | 24h | No | - |
| **27** | Exosomes Released from CaSR-Stimulated PMNs Reduce Ischaemia/Reperfusion Injury **Zhai** | S= 5% CO_2_, 95% N_2_ | 4h | n.s. | Not specified | **2 experiment groups**:  1x hypoxia 1x hypoxia + reoxygenation  S= Normal medium (DMEM containing  10% FBS) | 24h | No | - |
|  |  | C= 5% CO_2_, 95% air |  |  |  |  |  |  |  |
| **28** | MiR-21-5p-expressing bone marrow mesenchymal stem cells alleviate myocardial ischemia/reperfusion injury by regulating the circRNA_0031672/miR-21-5p/programmed cell death protein 4 pathway **Zhang** | S= 5% CO_2_, 1% O_2_, 94% N_2_  C= 5% CO_2_, 95% air | 12h | Humidified incubator | RPMI 1640 medium or Dulbecco’s  modified Eagle’s medium supplemented with 10%  FBS | S= 5% CO_2_ | 0 to 48h | No | - |

Table S 1, **Ischemia and Reperfusion Stress Treatments**. This table comprises all conditions used to mimic ischemia and reperfusion in the in vitro co-culture systems. Methods used are categorised as mimicking ischemia or reperfusion as stated by the authors. **ACM**= adult cardiomyocyte, **BM-MSC**= bone marrow-derived mesenchymal stem cells, **C**=control condition, **CM**= cardiomyocyte, **CSC**= cardiac stem cell, **DMEM**= Dulbecco's Modified Eagle Medium, **EGM-2**= endothelial cell growth medium-2, **FBS**= foetal bovine serum, **HEPES**= 4-(2-hydroxyethyl)-1-piperazineethanesulfonic acid, **hMADS**= human multipotent adipose-derived stem cells, **HUVECs**= human umbilical vein endothelial cells, **IFN-gamma**= interferon-gamma, **IMDM**= Iscove's Modified Dulbecco's Medium, **S**= stress condition, **SVP**= saphenous vein-derived pericytes, **NA**= not applicable, **n.s**.= not stated, **RPMI 1640** **medium**= Roswell Park Memorial Institute medium 1640, **PFA**= paraformaldehyde, **P/S**= penicillin/streptomycin

| **Cell Types and Culture Systems** | | | | | | |
| --- | --- | --- | --- | --- | --- | --- |
| **#** | **Publication Title and First Author** | **Cell Types** | **Species** | **Rationale for combining different species** (if applicable) | **Culture System** | **Material for in vitro culture** |
| **1** | „Three-Dimensional Paper-Based Model for Cardiac Ischemia”, **Mosadegh** | - Primary rat neonatal cardiomyocytes - Cardiac rat fibroblasts - 3T3 fibroblasts | - Rat (CMs, CFs) - Mouse (3T3) | Yes: Low availability of cardiac fibroblasts (from rat heart); therefore additional usage of 3T3 | Co-culture in layers: Cells in hydrogel-cell suspension applied on hydrophilic zones (separated through wax) on Whatman-Paper – papers were stacked | - Matrigel (for cell suspension) - Whatman 114 chromatography paper with wax print - Delrin holder (container for stacked papers) |
| **2** | “Transcriptome profiling of 3D co-cultured cardiomyocytes and endothelial cells under oxidative stress using a photocrosslinkable hydrogel system”,  **Yue** | - Primary rat neonatal cardiomyocytes (2-day old Sprague-Dawley rats) - hiPSCs (🡪 iECs) | - Rat (CMs) - Human (iECs) | Yes and No: questionable maturity and functionality of human iCMs and the better characterization of neonatal rat CM  **Advantage**: mRNAs were distinguishable and assignable to the respective cell types 🡪 detailed transcriptome analysis | Co-culture (without cell-cell interactions, 3D)   - CMs + iECs (4:1) | - Photocross-linkable 4-arm PEGACRL/4-arm PEG-ACRL-RGD 3D hydrogel |
| **3** | “Development of a Cell Co-Culture Model to Mimic Cardiac Ischemia/Reperfusion In Vitro”,  **Li** | - Primary murine coronary artery endothelial cells - Adult mouse cardiomyocytes | - Mouse | NA | Co-culture (Transwell):   - ECs + CMs | - Transwell-inserts for three different distances to the cells in the well below |
| **4** | „Modelling myocardial ischemia/reperfusion injury with inflammatory response in human ventricular cardiac organoids“,  **Zhang** | - hiPSC line ZB11AOF (🡪 iCMs) - THP-1 cells - MYL2-Venus human embryonic stem cells reporter line | - Human | NA | Organoid Culture (ventricular cardiac organoids from hiPSCs containing CMs, ECs, and fibroblasts)  Additionally: Organoid culture + THP-1 cells | - Ultra-low-attachment 96-well plates |
| **5** | “Development of an In Vitro Cardiac Ischemic Model Using Primary Human Cardiomyocytes”, **Hafez** | - Primary human cardiomyocytes - Human sternal bone marrow MSCs | - Human | NA | Co-Culture (direct, 2D):   - MSCs + CMs   (MSCs were added to CMs after hypoxia and reoxygenation phase) | - Plate |
| **6** | “Human CardioChimeras: Creation of a Novel “Next-Generation Cardiac Cell”,  **Firouzi** | Chimera (hCC) production:   - ckit+ cardiac interstitial cells (cCIC) and - Human MSCs from human neonatal postmortem cardiac tissue   For Co-culture:   - Neonatal rat cardiomyocytes | - Human (cCIC, MSC, chimeric cells) - Rat (neonatal rat cardiomyocytes) | None | Co-culture (direct, 2D):   - hCC + CMs   (Stem cells (control) or cardiochimera cells added at ratio 1:5 after stress treatment of neonatal rat CMs) | - Plate |
| **7** | “Combined Intramyocardial Delivery of Human Pericytes and Cardiac Stem Cells Additively Improves the Healing of Mouse Infarcted Hearts Through Stimulation of Vascular and Muscular Repair”,  **Avolio** | - Human cardiac stem cells (CSC) - Human saphenous vein-derived pericytes (SVP) | - Human | NA | Co-culture (direct and Transwell, 2D):   - CSCs + SVPs | - Plate - Transwell |
| **8** | “Bone marrow‑derived mesenchymal stem cells rescue injured H9c2 cells via transferring intact mitochondria through tunneling nanotubes in an in vitro simulated ischemia/reperfusion model”, **Han** | - Primary bone marrow-MSC from GFP-expressing Sprague Dawley rats - H9C2 rat ventricular cell line | - Rat | NA | Co-culture (direct, 2D):   - H9C2 + BM-MSCs   (BM-MSCs added in ratio 1:1 after hypoxia during reoxygenation) | - Plate |
| **9** | “Apoptosis-Resistant Cardiac Progenitor Cells Modified With Apurinic/Apyrimidinic Endonuclease/Redox Factor 1 Gene Overexpression Regulate Cardiac Repair After Myocardial Infarction”,  **Aonuma** | - Neonatal rat ventricular myocytes (NRVMs) - Sca-1+ murine cardiac progenitor cells (CPCs) 🡪 Modified to overexpress APE1 | - Rat - Mouse | None | Co-culture (direct, 2D):   - NRVMs + CPCs   (APE-1-CPCs or control CPCs were cultured with NRVMs at ratio 3:1) | - Plate |
| **10** | “Dedifferentiation, Proliferation and Redifferentiation of Adult Mammalian Cardiomyocytes after Ischemic Injury”,  **Wang** | - Murine adult cardiomyocytes (ACM) - Neonatal rat ventricular myocytes + cardiac fibroblasts (Sprague-Dawley) - Fetal cardiomyocytes (species not stated) | - Rat - Mouse | None | Co-culture (direct, 2D):   - ACM + NRVMs (1:20) - ACMs + Cardiac fibroblasts - ACMs + foetal CMs   Co-culture (Transwell):   - ACMs + NRVMs | - Plate - Transwell |
| **11** | “Human cardiac progenitor cell activation and regeneration mechanisms: Exploring a novel myocardial ischemia/reperfusion in vitro model”,  **Sebastiano** | - hiPSCs (🡪 iCM) - Human cardiac progenitor cells from human right atria appendage myocardial tissue | - Human | NA | Co-Culture (Transwell):   - iCMs + CPCs (CPCs added via transwell inserts at beginning of ischemic phase) | - Transwell |
| **12** | “Mesenchymal stem cells sense mitochondria released from damaged cells as danger signals to activate their rescue properties”,  **Mahrouf-Yorgov** | - Primary HUVECs - Human multipotent adipose-derived stem cells (hMADS) = MSC model - RL14 (human fetal ventricular cell line) | - Human | NA | Co-culture (2D, direct):   - MSC + HUVECs - MSC + RL14-CM   Co-culture (Transwell):   - MSC + HUVECs - MSC + RL14-CM   Co-culture ratio: 1:1 | - Plate - Transwell |
| **13** | “CB2 receptor-mediated effects of pro-inflammatory macrophages influence survival of cardiomyocytes” **Heinemann** | - Murine primary macrophages - Murine primary embryonic and adult cardiomyocytes (C57BL/6J WT mice) | - Mouse | NA | Co-culture (direct):   - 24-well: for images and cell count: 5*10^4 total CM + 1-5 *10^5 M1-Macrophages - 12-well plates for FACS: 1,5*10^5 CMs + 1-5 *10^5 M1-Macrophages | - Plate - Pre-coated coverslips Directly in plate, pre-coated coverslips. (not specified which coating) |
| **14** | “Silica Nanoparticles Actively Engage With Mesenchymal Stem Cells in Improving Acute Functional Cardiac Integration” **Popara** | - Human MSC from healthy bone marrow donors P 4-8 - H9C2 | - Human - Rat | None | Co-culture (direct):   - Human MSC + H9C2 (ratio 1:2) | - Plate |
| **15** | “Co-expression of Akt1 and Wnt11 promotes the proliferation and cardiac differentiation of mesenchymal stem cells and attenuates hypoxia/ reoxygenation-induced cardiomyocyte apoptosis“ **Bangdang** | - Rat MSC - H9C2 | - Rat | NA | Co-culture (Transwell):   - H9C2 + MSC with CMs in upper layer and MSC in lower layer (ratio 10:1) | - Transwell |
| **16** | “microRNA-423-3p exosomes derived From cardiac fibroblasts mediates the Cardioprotective effects of ischaemic post-conditioning” **Luo** | - Primary neonatal rat cardiac fibroblasts - H9C2 | - Rat | NA | Co-Culture (Transwelll):   - CF + H9C2  (no ratio specified) | - Transwell  (Pore Size 0,4µm (Corning)) |
| **17** | “Feasibility Analysis of Oxygen‑Glucose Deprivation‑Nutrition Resumption on H9c2 Cells In vitro Models of Myocardial Ischemia‑Reperfusion Injury” **Yang** | - Macrophages (species not stated) - H9C2 - HUVECS | - Human - Rat | Yes: Testing of protocols from literature – recreation/validation | Co-Culture (Transwell):   - CM + Macrophages + MSCs   (1 mio cells of CM in lower chamber, other cells (Macrophages, HUVECs) in upper chamber) | - Transwell |
| **18** | “Endothelial-Cell-Derived Human Secretory Leukocyte Protease Inhibitor (SLPI) Protects Cardiomyocytes against Ischemia/Reperfusion Injury” **Kongpol** | - Umbilical cord cells (EA.hy926) - H9C2 | - Human - Rat | Yes: Usage of cell lines because of “problems with primary cells” | Co-Culture (Transwell):   - EA.hy926 + H9C2 | - Transwell, - SLPI-overexpressing EA.hy926 cells (CM/EC ratio of 1:1) (CM/EC ratio of 1:3) |
| **19** | “Atorvastatin enhances the therapeutic efficacy of mesenchymal stem cells-derived exosomes in acute myocardial infarction via up-regulating long non-coding RNA H19” **Huang** | - HUVEC - H9C2 - Rat MSC Exosomes (?) | - Human - Rat | None | Co-Culture (Transwell):   - H9C2 + HUVECs | - Transwell   (First transwells, then MSC-exosomes added, then separately seeded in plate and hypoxia) |
| **20** | “Comparative Effect of MSC Secretome to MSC Co-culture on Cardiomyocyte Gene Expression Under Hypoxic Conditions *in vitro“* **Kastner** | - Human bone marrow derived MSC - Human primary CM | - Human | NA | Co-Culture (direct and Transwell):   - MSC + CM   (addition of 50000 cells MSC to CMCs. | - Transwell system 6µm (Greiner) - Plates |
| **21** | “Hypoxia-reoxygenation induces macrophage polarization and causes the release of exosomal miR-29a to mediate cardiomyocyte pyroptosis” **Wang** | - Bm derived macrophages from mice - Primary rat CM | - Rat - Mice | NA | Co-Culture (Transwell):   - CM+ M1 macrophages, - CM + M2 macrophages | - Transwell |
| **22** | **“**Dexmedetomidine attenuates myocardial ischemia-reperfusion injury in vitro by inhibiting NLRP3 Inflammasome activation” **Huang** | - Primary rat CF - Primary rat CMs | - Rat | NA | Co-Culture (direct):   - CF + CM (ratio of 1:3 (25% CFS)) | - Glass bottom dishes and plates |
| **23** | **“**Propofol pretreatment alleviates mast cell degranulation by inhibiting SOC to protect the myocardium from ischemia–reperfusion injury“ **Li** | - H9C2 - RBL-2H3 | - Rat | NA | Co-Culture (Transwell):   - H9C2 (lower chamber) + RBL-2H3 (upper chamber) | - Transwell |
| **24** | “Follistatin-like 1 promotes proliferation of matured human hypoxic iPSC-cardiomyocytes and is secreted by cardiac fibroblasts“ **Peters** | - hiPSC (🡪 iCFs, iCMs) - PBMCs | - Human | NA | Co-Culture (direct):   - iCMs + iCFs  (ratios: 80%:20%, 70%:30% and 50%:50%) | - Plates |
| **25** | Vascular smooth muscle cells activate PI3K/Akt pathway to attenuate myocardial ischemia/reperfusion-induced apoptosis and autophagy by secreting bFGF **Ye** | - H9C2 - Primary vascular smooth muscle cells (VSMCs) from rat | - Rat | NA | Co-culture method n.s. | - N.s. |
| **26** | Mitochondrial Respiratory Capacity is  Restored in Hibernating Cardiomyocytes  Following Co-Culture with Mesenchymal  Stem Cells Hocum Stone | - H9C2 - swine bone-marrow derived MSC | - Swine - Rat | NA | Co-culture (Transwell):   - H9C2 + bmMSC (ratio 2:1,5) | - Transwell cell culture inserts |
| **27** | Exosomes Released from CaSR-Stimulated PMNs Reduce Ischaemia/Reperfusion Injury **Zhai** | - Primary neonatal rat cardiomyocytes - Polymorphonucelar cells (PMNs) from whole blood (rat) | - Rat | NA | Co-culture (indirect)   - CMs + PMNs (ratio n.s.) | - Co-cultivation filters (Transwell) |
| **28** | MiR-21-5p-expressing bone marrow mesenchymal stem cells alleviate myocardial ischemia/reperfusion injury by regulating the circRNA_0031672/miR-21-5p/programmed cell death protein 4 pathway **Zhang** | - H9C2 - BMSCs (species n.s.) | - Rat | NA | Co-culture (indirect)   - H9C2 + BMSCs (ratio n.s.) | - Transwell insert |

Table S 2, **Cell types and culture systems used for the generation of in vitro MI model systems.** **ACM**= adult cardiomyocytes, **BM-MSC**= bone marrow-derived mesenchymal stem cells, **CF**= cardiac fibroblast, **CIC**= cardiac interstitial cells, **CM**= cardiomyocytes, **CPC**= cardiac progenitor cells, **EC**= endothelial cells, **hiPSC**= human induced pluripotent stem cells, **HUVECs**= human umbilical vein endothelial cells, **iCM**= induced cardiomyocytes, **iEC**= induced endothelial cells, **iCF**= induced cardiac fibroblasts, **MSC**= mesenchymal stem cells, **NA**= not applicable, **NRCM**= neonatal rat cardiomyocytes, **NRVM**= neonatal rat ventricular myocytes
